# A Regulatory Feedback between Plasmacytoid Dendritic Cells and Regulatory B Cells Is Aberrant in Systemic Lupus Erythematosus

**DOI:** 10.1016/j.immuni.2016.02.012

**Published:** 2016-03-15

**Authors:** Madhvi Menon, Paul A. Blair, David A. Isenberg, Claudia Mauri

**Affiliations:** 1 Centre for Rheumatology, Division of Medicine, University College London, 5 University Street, London WC1E 6JF, UK

## Abstract

Signals controlling the generation of regulatory B (Breg) cells remain ill-defined. Here we report an “auto”-regulatory feedback mechanism between plasmacytoid dendritic cells (pDCs) and Breg cells. In healthy individuals, pDCs drive the differentiation of CD19^+^CD24^hi^CD38^hi^ (immature) B cells into IL-10-producing CD24^+^CD38^hi^ Breg cells and plasmablasts, via the release of IFN-α and CD40 engagement. CD24^+^CD38^hi^ Breg cells conversely restrained IFN-α production by pDCs via IL-10 release. In systemic lupus erythematosus (SLE), this cross-talk was compromised; pDCs promoted plasmablast differentiation but failed to induce Breg cells. This defect was recapitulated in healthy B cells upon exposure to a high concentration of IFN-α. Defective pDC-mediated expansion of CD24^+^CD38^hi^ Breg cell numbers in SLE was associated with altered STAT1 and STAT3 activation. Both altered pDC-CD24^+^CD38^hi^ Breg cell interactions and STAT1-STAT3 activation were normalized in SLE patients responding to rituximab. We propose that alteration in pDC-CD24^+^CD38^hi^ Breg cell interaction contributes to the pathogenesis of SLE.

## Highlights

• pDCs induce the differentiation of Breg cells in an IFN-α-dependent manner
• Breg cells limit pDC-derived IFN-α in an IL-10-dependent mechanism
• pDCs are hyperactivated in SLE and fail to induce Breg cells
• Patients responding to rituximab display a normalized pDC-Breg cell interaction

## Graphical Abstract

**Figure:**
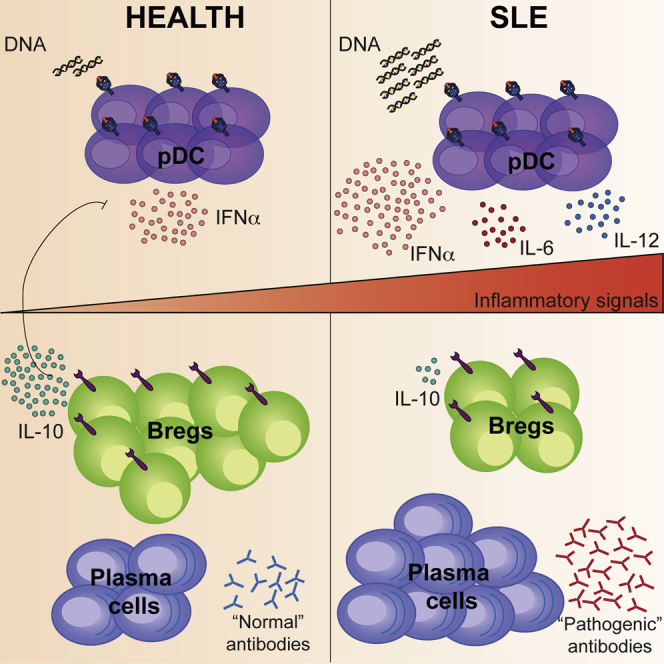

## Teaser

The signals required for Breg cell differentiation in humans are currently unknown. Mauri and colleagues show that plasmacytoid dendritic cells, via the provision of IFN-α, govern the differentiation of immature B cells into regulatory B cells that restrain inflammation.

## Introduction

Regulatory B (Breg) cells exhibit immunosuppressive functions via the release of IL-10, transforming growth factor (TGF)-β, and IL-35 and by induction of other regulatory cells (Mauri and Bosma, 2012, Mauri and Nistala, 2014). In healthy individuals, immature B cells have been shown to regulate T cell responses via the release of IL-10, suppressing T helper 1 (Th1) and Th17 cell differentiation, and by converting effector CD4^+^ T cells into FoxP3^+^CD4^+^ regulatory T (Treg) cells (Blair et al., 2010, Flores-Borja et al., 2013). In several autoimmune diseases, including SLE and rheumatoid arthritis (RA), Breg cells are functionally and numerically impaired (Blair et al., 2010, Flores-Borja et al., 2013). Signals required for the differentiation of human Breg cells remain poorly understood.

CD123^+^BDCA-2^+^ plasmacytoid dendritic cells (pDCs) are important drivers of innate and adaptive immune responses (McKenna et al., 2005, Reizis et al., 2011). pDCs rapidly produce large amounts of interferon alpha (IFN-α) upon toll-like receptor (TLR) activation during viral infections or in response to neutrophil extracellular traps (NETs) (Gilliet et al., 2008, Hoffmann et al., 2015, Garcia-Romo et al., 2011, Swiecki and Colonna, 2015). In SLE, neutrophils die upon exposure to SLE-derived anti-ribonucleoprotein antibodies and release NETs containing endogenous DNA as well as neutrophil proteins that enter pDC endocytic compartments and activate them to produce high amounts of IFN-α (Garcia-Romo et al., 2011, Lande et al., 2011). IFN-α stimulates multiple cell types, including natural killer (NK) cells, monocytes, myeloid DCs, and T cells, to release a variety of pro-inflammatory cytokines (McKenna et al., 2005). IFN-α produced by pDCs is pivotal in driving the maturation of B cells into plasmablasts (Jego et al., 2003, Poeck et al., 2004). pDCs can induce the differentiation of IL-10-producing T cells and FoxP3^+^ Treg cells to counterbalance inflammatory responses and to prevent excess inflammation (Ito et al., 2007, Moseman et al., 2004, Swiecki and Colonna, 2015).

IFN-α-induced gene signature, together with defects in B cell function, is considered the hallmark of SLE (Bennett et al., 2003, Obermoser and Pascual, 2010). In SLE, chronic activation of pDCs and other cells results in enhanced IFN-α and IFN-α/β receptor (IFN-α/βR) signaling on target cells (Rönnblom and Eloranta, 2013). Higher amounts of IFN-α production in SLE are associated with an accumulation of plasma cells, increased autoantibody, defective apoptotic cell clearance, and promotion of T-cell-dependent inflammation (Li et al., 2015, Pascual et al., 2006). In lupus-prone transgenic mice, transient depletion of pDCs prior to disease initiation reduces autoantibody, type I IFN signature, and kidney pathology compared to undepleted mice (Rowland et al., 2014). Similarly, IFN-α/βR blockade inhibits autoantibody production and protects young lupus-prone BXSB or MRL-Fas^lpr^ mice from disease, highlighting a role for pDCs in the disease initiation (Baccala et al., 2012). Furthermore, IRF8-deficient NZB mice, which lack pDCs, display a profound reduction in anti-nuclear, anti-chromatin, and anti-erythrocyte autoantibodies, as well as a significant reduction in kidney disease (Baccala et al., 2013). In addition, mice lacking E2-2, a transcription factor that regulates pDC development, display impaired pDC function, a dramatic reduction in anti-DNA autoantibody production, and glomerulonephritis as well as ameliorated disease (Sisirak et al., 2014).

Several studies have linked type I IFNs with an increase in IL-10 production by B cells (Matsumoto et al., 2014, Schubert et al., 2015). However, the role of pDCs and/or type I IFNs in determining whether a B cell becomes a Breg cell or an antibody-producing plasmablast remains unknown. Our data demonstrate that pDCs can generate plasmablasts that co-express IL-10, IL-6, and TNF-α and release antibody, as well as CD24^+^CD38^hi^ Breg cells. CD24^+^CD38^hi^ Breg cells provided negative feedback and restrained excessive IFN-α production by pDCs via IL-10 release. In SLE, pDCs failed to induce the differentiation of CD24^+^CD38^hi^ Breg cells but promoted antibody production. Similarly, in vitro stimulation of healthy B cells with high concentrations of IFN-α failed to expand CD24^+^CD38^hi^ Breg cells but promoted plasmablast differentiation. In comparison to healthy controls, immature B cells from SLE patients displayed altered STAT1 and STAT3 activation and expressed higher amounts of TNF-α and IL-6 than IL-10. Of note, newly repopulated immature B cells in SLE patients responding to rituximab showed normalized expression of STAT1 and STAT3 and could differentiate into CD24^+^CD38^hi^ Breg cells.

## Results

### pDCs Expand CD24^+^CD38^hi^ Breg Cells in Healthy Individuals

**Figure 1:**
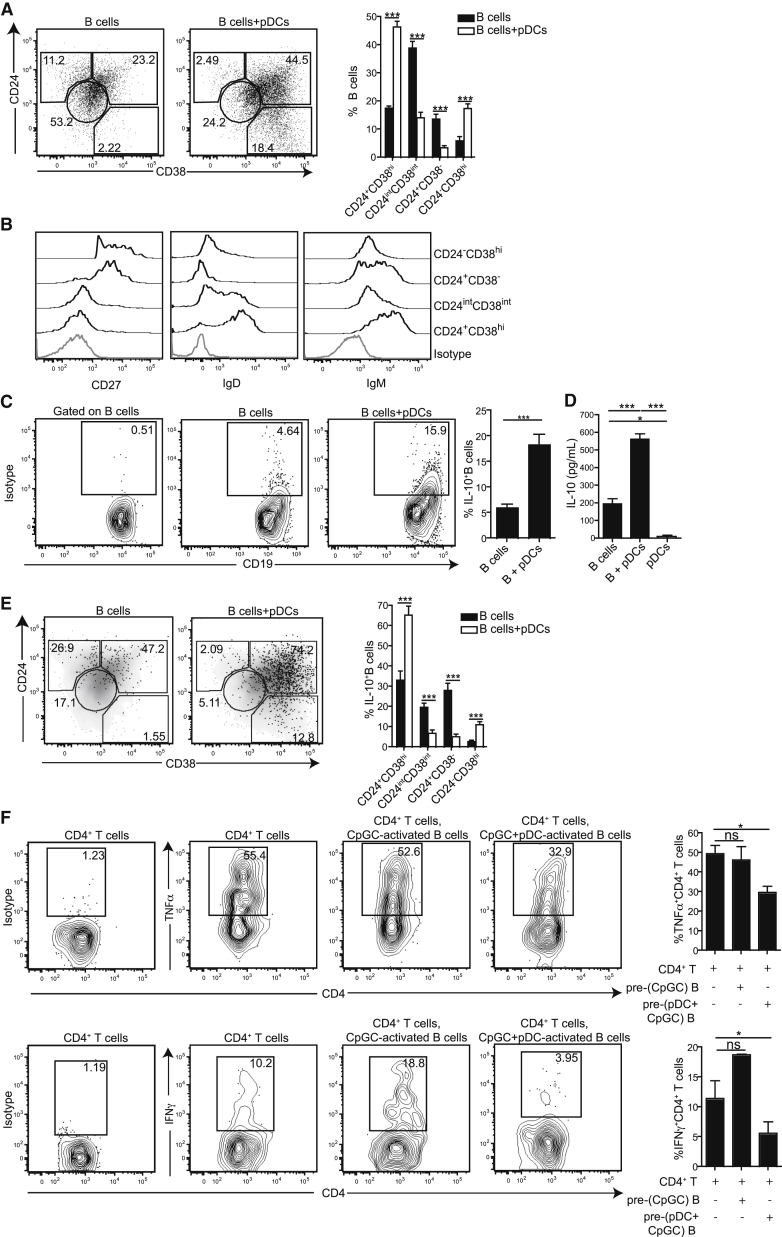
pDCs Expand Regulatory B Cells in Healthy Individuals (A) Representative flow cytometry plots and bar chart show frequency of CD24^+^CD38^hi^, CD24^int^CD38^int^, CD24^+^CD38^−^, and CD24^−^CD38^hi^ B cell subsets after 72 hr culture of B cells with CpGC or CpGC and autologous pDCs (n = 10). (B) Representative histograms show CD27, IgD, and IgM expression on B cell subsets after culture with autologous pDCs and CpGC. (C) Representative flow cytometry plots and summary bar chart displaying the frequency of IL-10^+^ B cells in B cell co-culture with CpGC and autologous pDCs (n = 18). (D) Bar chart shows IL-10 concentration in the supernatants of CpGC-stimulated B cells and autologous pDC co-cultures (n = 18). (E) Representative flow cytometry plots overlaying IL-10^+^ B cells (black dots) on total B cells (gray density plot) after B cell and pDC co-culture with CpGC, and bar chart showing percentage IL-10^+^ B cells within B cell subsets (n = 10). (F) Representative flow cytometry plots and bar charts showing TNF-α or IFN-γ expression by anti-CD3-stimulated CD4^+^CD25^−^ T cells after co-culture with autologous CD19^+^ B cells re-isolated from pre-cultures with CpGC or with CpGC and autologous pDCs (n = 18). Data are expressed as mean ± SEM. See also Figures S1 and S2.

To test whether pDCs could generate Breg cells, as well as plasma cells as previously reported (Jego et al., 2003), we co-cultured negatively isolated healthy B cells with autologous pDCs for 3 days with CpGC, a TLR9 agonist that stimulates both pDCs and B cells (Hartmann et al., 2003). Figures S1A and S1B show the purity of isolated B cells and pDCs. In addition to inducing the differentiation of CD24^−^CD38^hi^ B cells (plasmablasts), stimulation with pDCs and CpGC induced an expansion of CD24^+^CD38^hi^ B cells (Figure 1A) (comprising CD24^hi^CD38^hi^ and CD24^int^CD38^hi^ B cells as shown in Figure S1C). Of note, both CD24^hi^CD38^hi^ and CD24^int^CD38^hi^ B cells were IgM^hi^, IgD^hi^, and CD27^−^, characteristic of B cells at an immature stage of development (Figures S1D). Further phenotypical analysis confirmed that the pDC-expanded plasmablasts were CD27^+^, IgD^−^, and IgM^lo^, whereas pDC-expanded CD24^+^CD38^hi^ B cells were also CD27^−^, IgD^hi^, and IgM^hi^ (Figure 1B).

In addition to expanding B cells with an immature phenotype that has previously been associated with Breg cells (Blair et al., 2010), pDCs induced a 4-fold enrichment of IL-10-producing B cells compared to B cells stimulated with CpGC alone (Figure 1C). An expansion of IL-10-producing B cell numbers was observed even at low ratios (up to 1:50 pDCs:B cells) (Figure S1E). Analysis of the co-culture supernatants confirmed significantly increased amounts of IL-10 (Figure 1D). IL-10^+^ B cells generated by pDCs expressed higher Ki67 than IL-10^+^ B cells generated on stimulation with CpGC alone (Figure S1F). The majority of IL-10^+^ B cells induced by pDCs were CD24^+^CD38^hi^ B cells, with a smaller percentage residing within the plasmablast population, also previously ascribed with regulatory capacity (Figure 1E; Matsumoto et al., 2014). The majority of IL-10^+^ B cells were CD27^−^CD38^hi^ B cells, with a smaller percentage found within the CD27^+^CD38^hi^ population (Figure S1G). CpGC-stimulated pDCs displayed an enhanced ability to expand IL-10^+^ B cells compared to anti-CD3-stimulated CD4^+^ T cells, R848-stimulated monocytes, or poly(I:C)-stimulated BDCA-3^+^ conventional DCs (cDCs), all of which only marginally expanded IL-10^+^ B cells (Figures S2A–S2D).

To confirm that pDCs induce the differentiation of B cells with suppressive capacity, B cells were cultured alone with CpGC or with pDCs and CpGC for 48 hr. B cells were then re-isolated and co-cultured with autologous anti-CD3-stimulated CD4^+^CD25^−^ T cells for 72 hr. Whereas B cells re-isolated from pDC-B cell co-cultures suppressed the differentiation of IFN-γ^+^CD4^+^ T cells and TNF-α^+^CD4^+^ T cells, B cells re-isolated after culture with CpGC alone were unable to inhibit the differentiation of IFN-γ^+^ or TNF-α^+^CD4^+^ T cells (Figure 1F). Thus, TLR9-activated pDCs expand the frequency of suppressive IL-10^+^CD24^+^CD38^hi^ B cells and to lesser extent IL-10^+^CD24^−^CD38^hi^ B cells.

### Immature B Cells Are the Precursors of pDC-Induced Breg Cells

**Figure 2:**
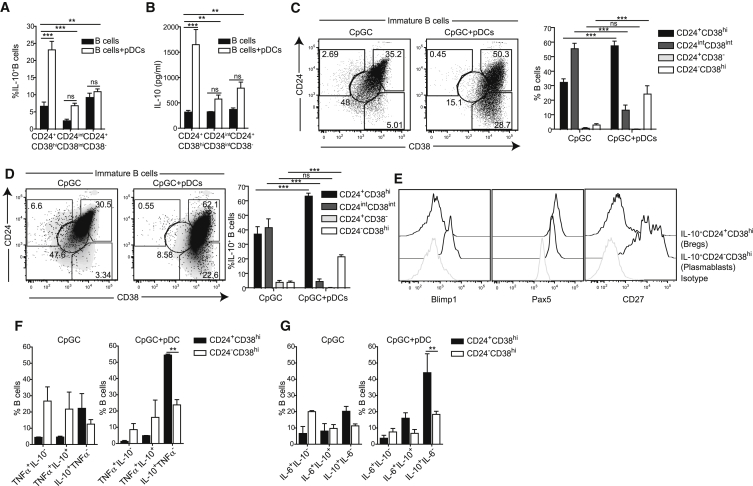
pDCs Expand CD24^+^CD38^hi^ Breg Cells within the Immature B Cell Subset in Healthy Individuals (A) Bar chart shows the frequency of IL-10^+^ B cells within FACS-sorted CD24^+^CD38^hi^ (immature), CD24^int^CD38^int^, and CD24^+^CD38^−^ B cell subsets, after culture with CpGC or CpGC and autologous pDCs for 72 hr (n = 4). (B) Bar chart shows IL-10 concentration in the culture supernatants from (A). (C) Representative flow cytometry plots and bar chart show frequency of CD24^+^CD38^hi^, CD24^int^CD38^int^, CD24^+^CD38^−^, and CD24^−^CD38^hi^ B cells after culture of immature B cells with CpGC or CpGC and autologous pDCs for 72 hr (n = 4). (D) Representative flow cytometry plots showing IL-10^+^ B cells (black dots) after culture of immature B cells with CpGC or with CpGC and autologous pDCs for 72 hr overlaid on a plot depicting CD24 and CD38 expression by CD19^+^ B cells (gray density plot). Bar charts show the frequency of IL-10^+^ B cells within the immature B-cell-derived B cell subsets (n = 4). (E) Representative histograms displaying Blimp1, Pax5, and CD27 expression on IL-10^+^CD24^+^CD38^hi^ and IL-10^+^CD24^−^CD38^hi^ B cell subsets after culture with autologous pDCs and CpGC. (F and G) Bar charts show the frequencies of (F) TNF-α^+^IL-10^−^, TNF-α^+^IL-10^+^, and TNF-α^−^IL-10^+^ B cells and of (G) IL-6^+^IL-10^−^, IL-6^+^IL-10^+^, and IL-6^−^IL-10^+^ B cells within the CD24^+^CD38^hi^ and CD24^−^CD38^hi^ B cell subsets after culture of immature B cells with CpGC or CpGC and autologous pDCs for 72 hr (n = 4). Data are expressed as mean ± SEM. See also Figure S3.

Next, we investigated whether the pDC-expanded CD24^+^CD38^hi^ Breg cells were derived directly from immature B cells or whether pDCs upregulated the expression of CD24 and CD38 on mature and memory B cells. CD24^hi^CD38^hi^ (immature), CD24^int^CD38^int^ (mature), and CD24^hi^CD38^−^ (memory) B cells were sorted by flow cytometry and stimulated with both CpGC and pDCs or with CpGC alone for 3 days (the purity of sorted B cell subsets is shown in Figure S3A). pDC-stimulated immature B cells produced significantly higher amounts of IL-10 compared to mature and memory B cells. Mature and memory B cells did not significantly upregulate IL-10 after co-culture with pDCs (Figures 2A and 2B). In response to CpGC and pDCs, CD24^int^CD38^int^ and CD24^+^CD38^−^ B cells preferentially differentiated into plasmablasts (Figure S3B), whereas immature B cells differentiated into CD24^+^CD38^hi^ and CD24^−^CD38^hi^ B cells (Figure 2C). Enrichment in IL-10-producing B cells was noted in both subsets after culture with CpG and pDCs (Figure 2D). Immature B cell-derived CD24^+^CD38^hi^ B cells were Blimp1^−^Pax5^+^IgD^hi^IgM^hi^, whereas plasmablasts were IgM^lo^IgD^−^Pax5^lo^Blimp1^+^ (Figure S3C), and IL-10^+^CD24^+^CD38^hi^ Breg cells were Blimp1^−^Pax5^+^CD27^−^ whereas IL-10^+^ plasmablasts were Blimp1^+^Pax5^lo^CD27^+^ (Figure 2E). Of interest, a similar effect of pDCs on CD24^+^CD38^hi^ Breg cells and IL-10 production was also observed when human cytomegalovirus (HCMV) lysates were used instead of CpGC (Figures S3D and S3E). These findings demonstrate that immature B cells are the direct precursors of pDC-induced CD24^+^CD38^hi^ Breg cells and plasmablasts expressing IL-10.

To investigate whether pDC-generated CD24^+^CD38^hi^ and CD24^−^CD38^hi^ B cells could both exhibit regulatory function, both B cell subsets were purified from the pDC:immature B cell co-culture and then co-cultured with anti-CD3 CD4^+^CD25^−^ T cells. Whereas CD24^+^CD38^hi^ B cells inhibited the differentiation of TNF-α^+^CD4^+^ T cells, CD24^−^CD38^+^ plasmablasts showed a negligible suppressive effect (Figure S3F). Plasmablasts purified from co-cultures of mature or memory B cells with pDCs did not show any suppressive effect (data not shown). Next, we measured whether pDC-generated CD24^+^CD38^hi^ B cells and plasmablasts expressed other cytokines. We observed that the vast majority of pDC-generated CD24^+^CD38^hi^ B cells expressed only IL-10 (Figures 2F and 2G); however, pDC-generated plasmablasts comprised similar proportions of IL-10, TNF-α, or IL-6 single expressing cells and TNF-α+IL-10 or IL-6+IL-10 co-expressing cells (Figures 2F and 2G). No IL-17, IFN-γ, IL-1β, IL-2, IL-23, or IL-21 was detected by intracellular staining in the pDC-generated CD24^+^CD38^hi^ B cells (Figure S3G).

### pDCs Promote Breg Cell Differentiation via IFN-α and CD40-CD40L

**Figure 3:**
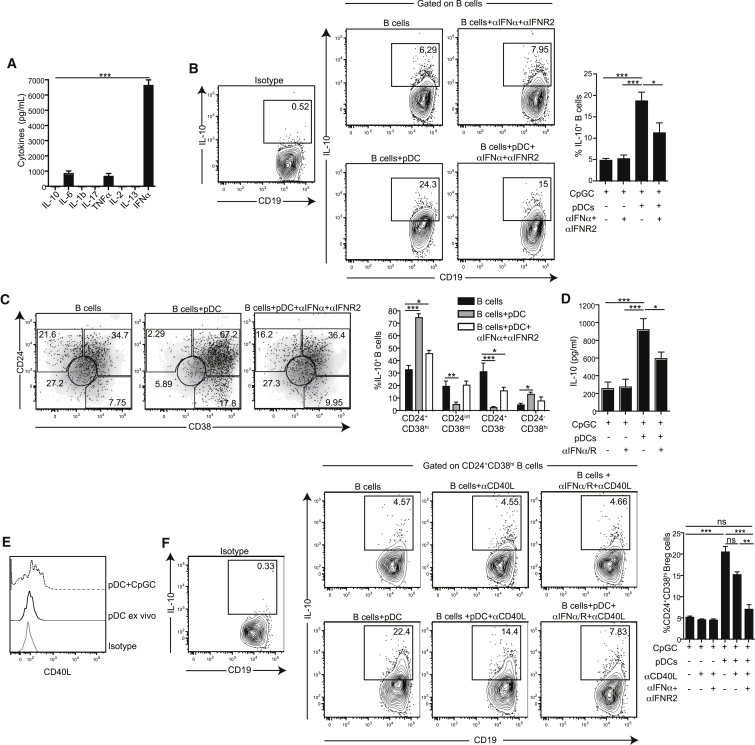
Healthy pDCs Expand CD24^+^CD38^hi^ Breg Cells via the Release of IFN-α and CD40 Engagement (A) Bar chart shows cytokines measured in the supernatants of pDCs stimulated with CpGC for 24 hr by cytometric bead array (CBA) (n = 8). (B) Representative flow cytometry plots and cumulative data show IL-10 expression by CD19^+^ B cells cultured with CpGC alone, or with pDCs and blocking antibodies to IFN-α and IFNAR2 (10 μg/mL mAb each) (n = 4). (C) Representative flow cytometry plots show IL-10^+^ B cells (black dots) after culture of B cells with CpGC alone or in the presence of pDCs and blocking antibodies to IFN-α and IFNAR2 overlaid on a plot depicting CD24 and CD38 expression by total CD19^+^ B cells (gray density plot). Bar chart show the frequency of IL-10^+^ B cells within the B cell subsets (n = 4). (D) Bar chart shows IL-10 measured in the supernatants in (A). (E) Representative histogram shows upregulation of CD154 expression on BDCA-2^+^CD123^+^ pDCs after stimulation with 1 μm CpGC (dotted line) compared to ex vivo (thick line). (F) Representative flow cytometry plots and cumulative data shows the frequency of CD24^+^CD38^hi^ Breg cells cultured alone or with pDCs and blocking antibodies to CD154 (10 μg/mL) or IFN-α and IFNAR2 (n = 4). Data are expressed as mean ± SEM. Data are representative of two independent experiments (B–F). See also Figure S4.

We next investigated the mechanism by which pDCs induce CD24^+^CD38^hi^ Breg cell differentiation. As previously shown (Siegal et al., 1999), the supernatants from CpGC-stimulated pDCs showed an abundance of IFN-α over other cytokines (Figure 3A). Neutralization of IFN-α and IFNα/βR2 (IFNR2) in pDC-B cell co-cultures resulted in a significant reduction in the frequency of IL-10^+^ B cells and IL-10^+^CD24^+^CD38^hi^ B cells and in the amount of IL-10 detected in the supernatants (Figures 3B–3D). Addition of exogenous IFN-α, IFN-β, or supernatants collected from CpGC-stimulated pDCs to TLR9-activated B cells resulted in an expansion of IL-10-producing CD24^+^CD38^hi^ Breg cells (Figures S4A–S4E).

CpGA and CpGB have been previously reported to differentially activate pDCs: CpGA stimulates pDCs to release IFN-α whereas CpGB induces the maturation of pDCs with minimal IFN-α production (Kerkmann et al., 2003). This provided us with a useful tool to test whether IFN-α produced by CpG-activated pDCs is necessary for B cell IL-10 production. pDCs were activated with CpGA or CpGB, washed, and then co-cultured with CpGB pre-activated B cells. pDCs and B cells stimulated with CpGC were used as a control. The results show that activation of pDCs with CpGA but not CpGB significantly expanded IL-10-producing B cells (Figure S4F).

CD24^hi^CD38^hi^ B cells have been reported to require CD40 signals to produce IL-10 (Blair et al., 2010). Expression of CD154 (CD40L) on pDCs was upregulated after CpGC stimulation (Figure 3E). Addition of anti-CD40L mAb to pDC-B cell co-cultures resulted in partial reduction of CD24^+^CD38^hi^ Breg cells, whereas simultaneous neutralization of IFN-α/IFN-R2 and CD40L further decreased CD24^+^CD38^hi^ Breg cell generation (Figure 3F). Blocking BAFF receptor in the pDC-B cell co-cultures had no effect on the differentiation of IL-10^+^ B cells. IL-10^+^ B cell induction was also independent of PD-1-PD-L1 or -PD-L2 interactions (Figures S4G–S4J), reported to be important in the differentiation of Treg cells (Francisco et al., 2009).

### pDC Activation Inversely Correlates with CD24^+^CD38^hi^ Breg Cell Frequency

**Figure 4:**
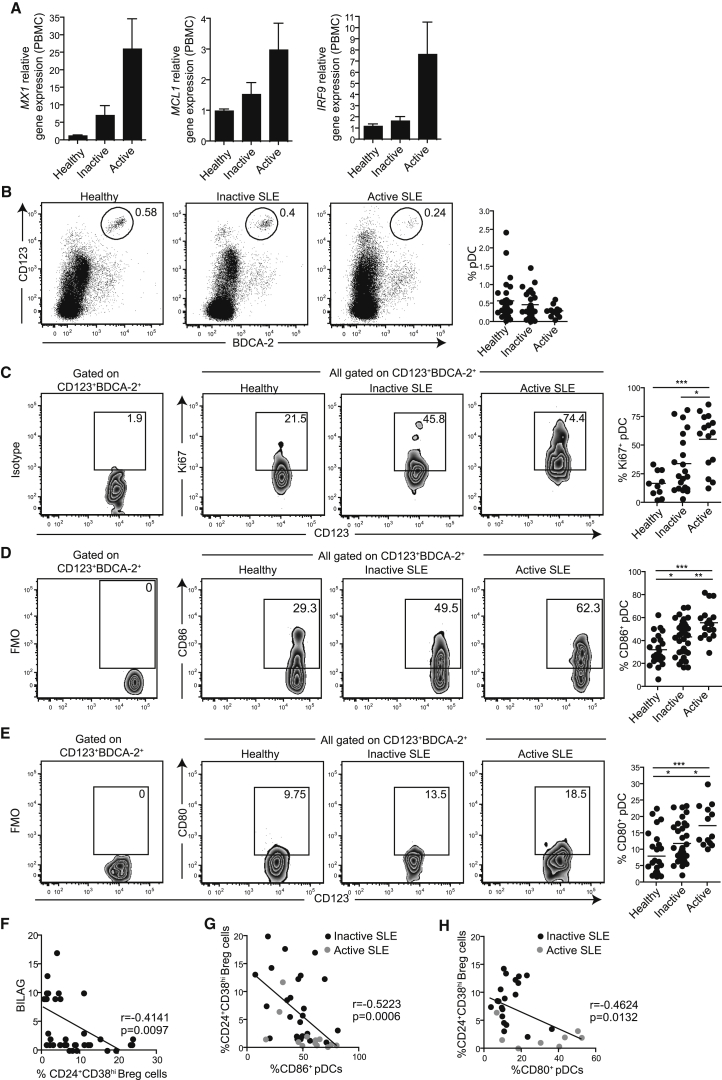
Type I IFN Signature and pDC Activation Status Are Elevated in SLE Patients and Vary with Disease Severity SLE patients were grouped based on disease severity as patients with either active (BILAG > 8) or inactive (BILAG < 8) disease. (A) Bar charts show the expression of genes *MX1*, *MCL1*, and *IRF9* on PBMCs from five healthy individuals, five inactive, and five active SLE patients. (B) Representative flow cytometry plots and cumulative data show the frequency of BDCA-2^+^CD123^+^ pDCs from 35 healthy, 31 inactive, and 15 active SLE PBMCs. (C) Representative flow cytometry plots and cumulative data show the frequency of Ki67^+^ pDCs from 11 healthy, 21 inactive, and 15 active SLE PBMCs. (D) Representative flow cytometry plots and cumulative data show the frequency of CD86^+^ pDCs from 23 healthy, 42 inactive, and 17 active SLE PBMCs. (E) Representative flow cytometry plots and cumulative data show the frequency of CD80^+^ pDCs from 27 healthy, 35 inactive, and 10 active SLE PBMCs. (F) Scatter plots show the relationship between BILAG scores and frequency of CD24^+^CD38^hi^ Breg cells in 38 SLE patients after CpGC stimulation of PBMCs for 72 hr. (G and H) Correlation plots show the relationship between frequency of CD24^+^CD38^hi^ Breg cells after CpGC stimulation of PBMCs for 72 hr and (G) CD86^+^ pDCs (n = 39) or (H) CD80^+^ pDCs (n = 28) for inactive (black dots) and active (gray dots) SLE patients. See also Figure S5 and Table S1.

Previous studies have reported that an elevated IFN-I gene signature correlates with disease severity (Bennett et al., 2003, Feng et al., 2006, Obermoser and Pascual, 2010). We also observed that SLE patients with active disease (BILAG > 8) displayed an elevated IFN-I signature measured by the expression of *MX1*, *MCL1*, and *IRF9*, compared to patients with inactive disease (BILAG < 8) and healthy individuals (Figures 4A). pDCs from active SLE patients displayed higher relative expression of *IFNA1* and *IRF7*, a transcription factor known to play a critical role in the induction of type I IFN (Honda et al., 2005), as well as *IL6* and *IL12B*, but not *IL12A*, compared to healthy individuals (Figure S5A). A significant increase in the amount of IFN-α was also detected in supernatants of CpGA-stimulated PBMCs from active SLE patients compared to healthy individuals (Figure S5B).

If pDCs induce CD24^+^CD38^hi^ Breg cells via IFN-α, then why are CD24^+^CD38^hi^ Breg cells reduced in SLE despite the presence of chronically activated pDCs and a type I IFN signature? To address this, we assessed whether there was a link between CD24^+^CD38^hi^ Breg cell frequency and the activation of pDCs ex vivo in SLE patients. Unlike previous studies showing a numerical deficiency in the number of pDCs (Blanco et al., 2001, Cederblad et al., 1998), the frequency of pDCs was unchanged in our cohort of patients (Figure 4B). The percentages of pDCs expressing Ki67, CD86, and CD80, hallmarks of TLR-induced pDC activation (McKenna et al., 2005), were significantly increased in SLE patients with active disease compared to inactive patients or healthy controls (Figures 4B–4E). Ex vivo expression of CD83, a DC maturation marker (Lechmann et al., 2002), was undetectable in pDCs (data not shown). In addition, CD24^+^CD38^hi^ Breg cell frequency inversely correlated with disease severity (Figures 4F), as well as with pDC activation status, measured by CD80 and CD86 expression, in active and inactive patients (Figures 4G and 4H).

SLE patients were also grouped for analysis based on the expression of the IRF-9 as a marker of an IFN signature. Figure S5C shows that patients with a higher IFN signature (IFN^hi^) have active disease, a higher frequency of activated pDCs (measured by the expression of CD80 and CD86), and a lower frequency of CD24^+^CD38^hi^ Breg cells compared to patients with a low IFN-gene signature (IFN^lo^). Similar results were obtained after segregating patients on MX1 and MCL1 expression levels (data not shown). Disease activity definitions and patient information are given in Table S1.

### SLE pDCs Failed to Promote Differentiation of CD24^+^CD38^hi^ Breg Cells

**Figure 5:**
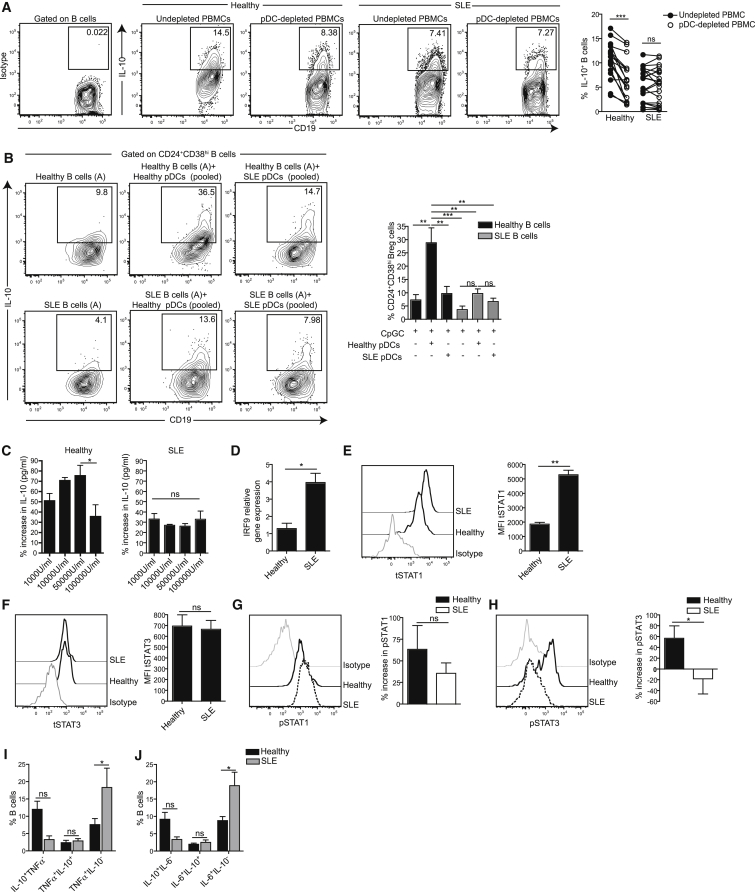
pDCs from SLE Patients Do Not Promote Breg Cell Expansion (A) Representative flow cytometry plots and cumulative data show the frequency of IL-10^+^ B cells in CpGC-stimulated pDC-depleted or undepleted PBMCs from 22 healthy individuals and 21 SLE patients. (B) Representative flow cytometry plots and bar graph show the frequency of IL-10^+^B cells after co-culture of healthy (n = 4) and SLE (n = 4) B cells with allogeneic-pooled CpGC-stimulated pDCs from healthy individuals or SLE patients as indicated; mean ± SEM. (C) Bar chart shows percentage increase in IL-10 concentration in supernatants collected from cultures of healthy (n = 4) or SLE (n = 4) B cells with CpGC and 1,000 U/mL, 10,000 U/mL, 50,000 U/mL, or 100,000 U/mL of IFN-α, compared to IL-10 concentration in supernatants taken from B cell cultures with CpGC alone. (D) Bar chart shows relative expression of IRF9 ex vivo by purified B cells isolated from healthy (n = 3) or SLE (n = 3) PBMCs. (E and F) Representative histograms and bar charts show MFI for (E) tSTAT1 and (F) tSTAT3 expression by healthy (n = 3) or SLE (n = 3) CD19^+^ B cells, detected ex vivo by FACS. Bar charts show percentage increase in MFI at 15 min (E) or 1 min (F) compared to time zero detected by FACS. (G and H) Representative histograms show expression of pSTAT1 (G) and pSTAT3 (H), by healthy (n = 3) and SLE (n = 3) CD24^+^CD38^hi^ B cells after in vitro stimulation of PBMCs with IFN-α (10,000 U/mL). (I and J) Bar charts show percentages of (I) IL-10^+^TNF-α^−^, TNF-α^+^IL-10^+^, and TNF-α^+^IL-10^−^ expressing CD24^+^CD38^hi^ B cells, or (J) IL-10^+^IL-6^−^, IL-6^+^IL-10^+^, and IL-6^+^IL-10^−^ expressing CD24^+^CD38^hi^ B cells measured by FACS after stimulation of healthy (n = 6) or SLE (n = 8) PBMCs with CpGC. Data are expressed as mean ± SEM. Data are representative of two independent experiments (D–H). See also Figure S6 and Table S1.

Next, we investigated the functional outcome of pDC-B cell interactions in SLE patients. Using ImageStream technology, we visualized multiple pDC-B cell interactions in CpGC-stimulated PBMCs. No differences were found in the numbers of pDC-B cell conjugates in healthy and SLE PBMCs (Figure S6A). However, although healthy B cells interacting with pDCs expressed IL-10, SLE B cells interacting with pDCs did not (Figure S6B). Because patients with SLE are often lymphopenic (Rivero et al., 1978), we were unable to obtain sufficient pDCs from SLE PBMCs to assess whether they induce Breg cells in co-cultures with autologous B cells. To circumvent this problem, we depleted pDCs from healthy or SLE PBMCs and measured the effect that pDC depletion had on the expansion of IL-10^+^ B cells or B cell subsets. (Figure S6C confirms the depletion of pDCs from PBMCs by flow cytometry sorting.) It is noteworthy that, although SLE patients displayed lymphopenia, the pDC:B cell ratios in healthy and SLE PBMCs were comparable (data not shown). Frequencies of IL-10^+^ B cells were significantly reduced in pDC-depleted healthy PBMCs but not in SLE PBMCs (Figure 5A). However, depletion of pDCs from CpGC-stimulated healthy and SLE PBMCs resulted in a significant reduction in the frequency of both CD24^+^CD38^hi^ B cells and plasmablasts, compared to undepleted PBMCs (Figure S6D). IgG production by pDC-depleted PBMCs was decreased in both healthy and SLE PBMCs, whereas IgM levels were unaffected (Figures S6E and S6F). Of note, no significant differences were observed in B cell subset frequencies ex vivo in healthy and SLE PBMCs (Figure S6G).

Our results suggest that the lack of CD24^+^CD38^hi^ Breg cell induction observed in SLE could be the consequence of defective stimulation by pDCs. To evaluate this, healthy B cells were co-cultured either with pooled allogeneic healthy pDCs or with pDCs from SLE patients and stimulated with CpGC, and then CD24^+^CD38^hi^ Breg cell induction was assessed by flow cytometry. pDCs from healthy individuals induced a 5-fold expansion of CD24^+^CD38^hi^ Breg cells, whereas less than a 2-fold expansion was induced by SLE pDCs, indicating that pDCs are defective in SLE (Figure 5B). It is also possible that SLE B cells are refractory to stimulation by pDCs; upon stimulation with CpGC, healthy pDCs induced a partial increase of CD24^+^CD38^hi^ Breg cells from SLE B cells, whereas SLE pDCs failed to induce CD24^+^CD38^hi^ Breg cells from SLE B cells (Figure 5B). TLR9 gene expression ex vivo was comparable between healthy and SLE B cells (Figure S6H). These data suggest that both pDCs and B cells are defective in patients with SLE.

### SLE B Cells Display an Altered Response to IFN-α Stimulation

To investigate whether in vivo exposure of B cells to high levels of IFN-α released by chronically activated pDCs could explain the reduced frequency of IL-10^+^ B cells in SLE, B cells were stimulated with CpGC and increasing doses of IFN-α. Healthy B-cell-derived IL-10 production increased up to 50,000 U/mL of IFN-α, but at higher concentrations IL-10 production was significantly reduced (Figure 5C). IFN-α induced only low levels of IL-10 production from SLE B cells, which did not increase with higher IFN-α concentrations (Figure 5C). Culture with increasing IFN-α concentrations, up to 50,000 U/mL, also increased the frequency of CD24^+^CD38^hi^ B cells and CD24^+^CD38^hi^ Breg cells in healthy, but not in SLE, B cell cultures (Figures S7A and S7B). In contrast, stimulation of both healthy and SLE B cells with increasing IFN-α concentrations continued to promote plasmablast differentiation, but not their expression of IL-10, even at the highest concentrations (Figures S7C and S7D).

Engagement of IFN-α/βR by type I IFN results in the recruitment, phosphorylation, and nuclear translocation of signal transducer and activation of transcription proteins (STATs) and other transcription factors (Ivashkiv and Donlin, 2014). In particular, IFN-α/βR signaling results in the recruitment of IRF9 and STAT1, which are involved in the transcription of interferon-stimulated genes (ISGs) and anti-nuclear antibody production in a mouse model of SLE (Thibault et al., 2008), and STAT3, which is involved in the transcription of the IL-10 locus (Benkhart et al., 2000). Ex vivo, the expression of IFR9 and total STAT1 (tSTAT1), but not total STAT3, were significantly increased in B cells from SLE patients compared to healthy controls (Figures 5D–5F). In response to IFN-α, phosphorylation of STAT3, but not STAT1, was significantly decreased in SLE CD24^+^CD38^hi^ B cells compared to healthy CD24^+^CD38^hi^ B cells (Figures 5G and 5H). No differences between healthy and SLE were identified in the phosphorylation of STAT1 or STAT3 in the other B cell subsets (data not shown). In SLE B cells, these changes in total STAT1 and phosphorylation of STAT3 were reflected in a reduced frequency of single IL-10-producing B cells and an increase in the expression of TNF-α and IL-6, compared to healthy controls after in vitro stimulation of PBMCs with CpGC to activate pDCs (Figures 5I and 5J).

### Healthy but Not SLE CD24^+^CD38^hi^ Breg Cells Suppress pDC-Derived IFN-α via IL-10

**Figure 6:**
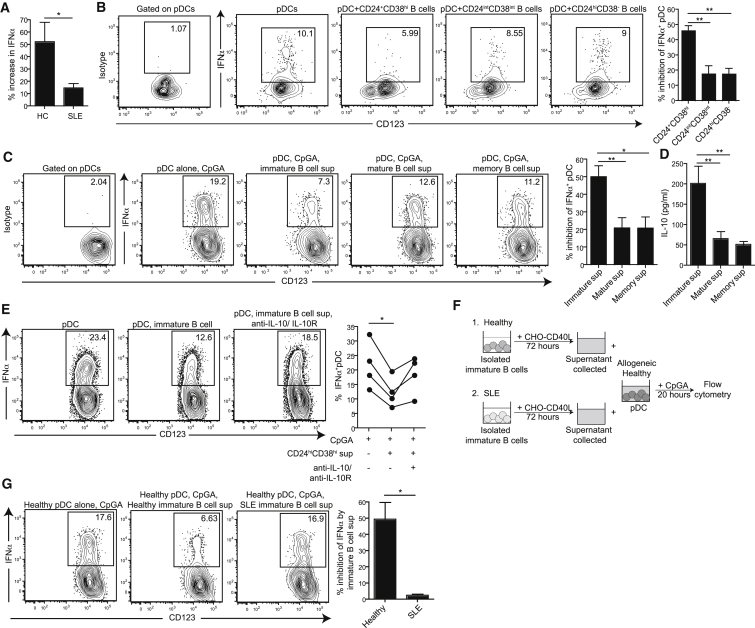
Healthy but Not SLE CD24^+^CD38^hi^ Breg Cells Suppress pDC IFN-α via IL-10 (A) Bar chart shows percentage increase in IFN-α concentration in the supernatants of CpGC-stimulated healthy (n = 7) or SLE (n = 7) PBMCs that have been depleted of CD19^+^CD24^+^CD38^hi^ B cells, compared to IFN-α concentration in supernatants of CpGC-stimulated undepleted PBMCs. (B) Representative flow cytometry plots show IFN-α expression by CpGA-stimulated healthy pDCs, after co-culture with purified autologous mCD40L (1 μg/mL) pre-stimulated CD19^+^CD24^+^CD38^hi^ (immature), CD19^+^CD24^int^CD38^int^ (mature), or CD19^+^CD24^+^CD38^−^ (memory) B cells. Bar chart shows the percentage inhibition of IFN-α^+^ pDCs (n = 3). (C) Representative flow cytometry plots show IFN-α expression by CpGA-stimulated healthy pDCs after co-culture with supernatants isolated from flow cytometry-sorted CD154-CHO cell-stimulated immature, mature, and memory B cells. Bar charts show the mean percentage inhibition of pDC IFN-α expression (n = 12). (D) Bar charts show IL-10 measured in the supernatants collected from B cell cultures in (C). (E) Representative flow cytometry plots show IFN-α expression by CpGA-stimulated healthy pDCs after co-culture with supernatants isolated from FACS-sorted CD154-CHO cell-stimulated immature B cells in the presence or absence of IL-10/IL-10R blockade. Scatter plot shows IFN-α expression for four individuals with or without supernatants and IL-10 blockade. (F) Schematic depicting experiment in (G). (G) Representative flow cytometry plots show IFN-α expression by CpGA-stimulated healthy pDCs after co-culture with supernatants isolated from FACS-sorted healthy or SLE CD154-CHO cell-stimulated immature B cells. Bar chart shows the mean percentage inhibition of pDC IFN-α expression (n = 3). Data are expressed as mean ± SEM. See also Figure S7 and Table S1.

IL-10 is known to inhibit pDC-derived IFN-α production in TLR-activated PBMCs (Duramad et al., 2003). To investigate whether IL-10^+^ Breg cells control excessive IFN-α production, we assessed the effect of CD24^+^CD38^hi^ B cell depletion from healthy and SLE PBMCs on IFN-α production by pDCs. CD24^+^CD38^hi^ B cells were depleted from PBMCs, which were then stimulated with CpGC for 20 hr (Figure S7E confirms the depletion of CD24^+^CD38^hi^ B cells from PBMCs by flow cytometry sorting). Depletion of CD24^+^CD38^hi^ B cells from healthy PBMCs resulted in a 50% increase in IFN-α production whereas depletion of CD24^+^CD38^hi^ B cells from SLE PBMCs resulted in only a 10% increase in IFN-α production (Figure 6A). These results suggest that CD24^+^CD38^hi^ B cells control pDC IFN-α production in healthy individuals but not in SLE patients.

To test whether healthy CD24^+^CD38^hi^ Breg cells can directly suppress IFN-α^+^ pDCs, sorted healthy immature, mature, and memory B cells were pre-stimulated with soluble CD40L for 48 hr and then cultured with autologous pDCs and CpGA for 20 hr. CD24^+^CD38^hi^ B cells displayed significantly greater suppression of pDC IFN-α expression than other subsets (Figure 6B). Culture of CpGA-stimulated pDCs with supernatants from CD40L-stimulated healthy immature B cells, but not other subsets, induced a similar degree of suppression of IFN-α (Figure 6C). Addition of anti-IL-10/IL-10R mAb to pDCs cultured with supernatants from CD40-activated immature B cells, which displayed the highest concentration of IL-10, restored IFN-α expression by pDCs (Figures 6D and 6E).

Because of the difficulties in obtaining sufficient pDC numbers from SLE patients, we could not assess whether SLE CD24^+^CD38^hi^ B cells could suppress autologous pDC responses directly. As an alternative, we compared whether supernatants isolated from healthy or SLE immature B cells were able to inhibit IFN-α production by allogeneic healthy pDCs (Figure 6F). Supernatants from CD40L-stimulated healthy immature B cells, but not from SLE immature B cells, suppressed IFN-α production by allogeneic healthy pDCs (Figure 6G). These results highlight that Breg cells can regulate IFN-α production by pDCs in healthy individuals but not in SLE patients.

### Rituximab Therapy Corrects pDC-Breg Cell Interactions

**Figure 7:**
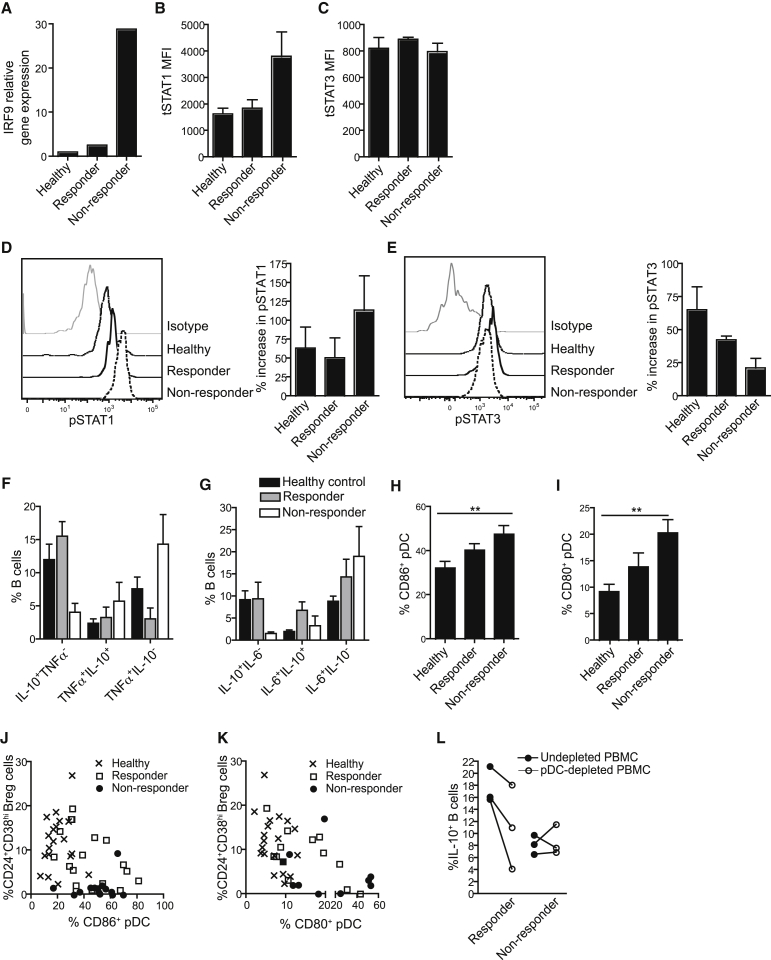
CD24^+^CD38^hi^ Breg Cell Function and pDC Activation Are Normalized in Patients Responding to Rituximab Therapy after B Cell Repopulation (A) Bar chart shows relative expression of IRF9 by purified B cells isolated from healthy individuals (Healthy), SLE patients responding to rituximab treatment (Responder), and SLE patients who don’t respond to rituximab treatment (Non-responder). (B and C) Bar charts show MFI for (B) tSTAT1 and (C) tSTAT3 expression ex vivo by B cells from healthy, responder, and non-responder (n = 3), detected by FACS. (D and E) Histograms and bar charts show the expression of (D) pSTAT1 and (E) pSTAT3 by CD24^hi^CD38^hi^B cells from healthy, responders, and non-responders, after in vitro stimulation of PBMCs with IFN-α (10,000 U/mL). Bar charts show percentage increase in MFI at 15 min (D) and 1 min (E) compared to time zero detected by flow cytometry (n = 3). (F and G) Bar charts show percentages of (F) IL-10^+^TNF-α^−^, TNF-α^+^IL-10^+^, and TNF-α^+^IL-10^−^ expressing CD24^+^CD38^hi^ B cells, or (G) IL-10^+^IL-6^−^, IL-6^+^IL-10^+^, and IL-6^+^IL-10^−^ expressing CD24^+^CD38^hi^ B cells measured by flow cytometry after stimulation of healthy (n = 6), responder (n = 6), or non-responder (n = 5) PBMCs with CpGC for 72 hr. (H and I) Bar charts show the frequency of (H) CD86^+^ pDCs in PBMCs from healthy (n = 23), responders (n = 30), and non-responders (n = 20), and (I) CD80^+^ pDCs in PBMCs from healthy (n = 23), responders (n = 30), and non-responders (n = 20). (J–L) Scatter plots show relationship of the frequencies of CD24^hi^CD38^hi^ Breg cells and (J) CD86^+^ pDCs in PBMCs of healthy (n = 19), responders (n = 18), and non-responders (n = 13), or (K) CD80^+^pDCs in the PBMCs from healthy (n = 19), responders (n = 13), and non-responders (n = 10). Scatter plot (L) shows the frequency of IL-10^+^ B cells in CpGC-stimulated pDC-depleted PBMCs or matched undepleted PBMCs for responders and non-responders (n = 3). Data representative of three independent experiments are expressed as mean ± SEM. See also Figure S7 and Table S1.

Patients treated with rituximab after B cell repopulation can be divided into two groups: those responding to rituximab (responders) and those not responding to rituximab (non-responders) therapy (Leandro et al., 2006). We investigated whether the newly repopulated B cells in responding patients could differentiate into Breg cells and whether this would be accompanied by a normalization of pDC function. Newly repopulated B cells in responders expressed IRF9 and tSTAT1 comparable to healthy controls, whereas non-responders displayed higher expression of IRF9 and tSTAT1 (Figures 7A and 7B). No differences were observed in the expression of tSTAT3 (Figure 7C). Upon stimulation with IFN-α, pSTAT1 expression increased and pSTAT3 decreased in CD24^+^CD38^hi^ B cells from non-responders compared to responders and healthy individuals (Figures 7D and 7E). Restoration of STAT3 phosphorylation in responding patients was reflected by normalization of the frequency of CD24^+^CD38^hi^ Breg cells, after in vitro stimulation of PBMCs with CpGC to activate pDCs. In contrast, non-responding patients showed an increase in expression of TNF-α and IL-6 and a reduction in CD24^+^CD38^hi^ Breg cells, compared to healthy controls and responders (Figures 7F and 7G).

In responding patients, the restoration of CD24^+^CD38^hi^ Breg cell function corresponded to a decrease in the activation of pDCs, as measured by CD80 and CD86 expression. In non-responding patients, the pDCs retained an activated phenotype (Figures 7H and 7I). Responders showed levels of Breg cells and pDC activation closer to healthy controls than non-responders (Figures 7J and 7K). In vitro depletion of pDCs from PBMCs of responders reduced the differentiation of IL-10^+^ B cells compared to undepleted PBMCs, indicating that the pDCs have reacquired the ability to induce CD24^+^CD38^hi^ Breg cells in responding patients. In non-responders, depletion of pDCs from PBMCs had no effect on the expansion of CD24^+^CD38^hi^ Breg cells (Figure 7L).

## Discussion

Experimental models of autoimmune diseases support a pivotal role for pro-inflammatory cytokines in the differentiation of Breg cells (Rosser et al., 2014, Yoshizaki et al., 2012). The effect that inflammatory stimuli have on the differentiation of human Breg cells remains unclear. Here we report that TLR9-activated pDCs directly interact with immature B cells and promote their differentiation into CD24^+^CD38^hi^ Breg cells as well as plasmablasts.

pDCs play a pleiotropic role in the immune system, controlling both the initiation of pro-inflammatory responses necessary to clear infection and the differentiation of regulatory cells (Swiecki and Colonna, 2015). Our data showing that pDCs are pivotal in the differentiation of CD24^+^CD38^hi^ Breg cells further strengthen the multi-functional role of pDCs in linking innate and adaptive immune responses. The expansion of IL-10-producing B cells and CD24^+^CD38^hi^ Breg cells was dependent on the release of an optimum amount of IFN-α by pDCs. This is in contrast to a previous study reporting that pDCs promote IL-10 production by B cells in an IFN-α-independent manner (Georg and Bekeredjian-Ding, 2012). Discrepancies between the two studies could be due to the usage of different TLR9 agonists: Georg and Bekeredjian-Ding (2012) used CpGB, rather than CpGC, to stimulate pDCs and test the involvement of type I IFN in the pDC-B cell interaction. As previously reported, CpGB induces negligible amount of IFN-α by pDCs and preferentially promotes their maturation (Kerkmann et al., 2003). The comparison of different TLR9 agonists in our study confirms that activation of pDCs with CpGC, but not CpGB, significantly expands IL-10-producing B cells via the release of IFN-α. Different concentrations of exogenous IFN-α added to B cells might also explain some of the differences reported. A role for IFN-α in the expansion of IL-10-producing B cells by pDCs cannot be entirely excluded without neutralization of IFN-α and IFN-α/βR in the pDC:B cell co-culture and this was not being tested in the study by Georg and Bekeredjian-Ding (2012).

Findings in a number of disease settings support a role for IFN-α-producing pDCs in the differentiation of CD24^+^CD38^hi^ Breg cells. In patients with human immunodeficiency virus (HIV) infection, IFN-α measured in the sera correlated with the disease severity and with increased number of CD24^hi^CD38^hi^ B cells in circulation (Malaspina et al., 2006). CD24^hi^CD38^hi^ Breg cell enrichment in patients infected with HIV correlated with the viral load (Siewe et al., 2013). In the same studies, the CD24^hi^CD38^hi^ Breg cells suppressed HIV-1-specific CD8^+^ T cell responses in an IL-10-dependent manner. Collectively, these results suggest that the IFN-α released by pDCs in response to HIV infection might promote the expansion of CD24^hi^CD38^hi^ Breg cells and the suppression of anti-viral immune response. Further evidence of a role for pDCs and IFN-α in the expansion of CD24^+^CD38^hi^ Breg cells is provided by data reporting an enrichment of IL-10^+^CD24^hi^CD38^hi^ B cells in multiple sclerosis (MS) patients responding to IFN-β therapy (Schubert et al., 2015). In this study, the authors showed that B-cell-deficient mice are resistant to the IFN-β therapy, which otherwise treats experimental autoimmune encephalitis in wild-type mice, suggesting that B cells mediate the immunosuppressive effects of IFN-β therapy.

We found that the concentration of IFN-α determines whether an immature B cell develops into a CD24^+^CD38^hi^ Breg cell or a plasmablast. At lower concentrations of IFN-α, B cells differentiate into both plasmablasts and CD24^+^CD38^hi^ Breg cells, whereas at higher concentrations, the B cell response is channeled toward plasma cell maturation. A similar inhibitory effect was reported in another study showing that whereas lower concentrations of IFN-α promote FoxP3 expression, a high concentration (100,000 U/mL) inhibits the expression of FoxP3 on T cells (Shibuya and Hirohata, 2005). Physiologically, the ability of moderate concentrations of IFN-α to induce both plasmablasts and CD24^+^CD38^hi^ Breg cells might reflect the fact that an immune response to an infection requires both antibody production to eliminate invading pathogens and the generation of regulatory cells to prevent chronic inflammation. IFN-α-supported plasmablast and CD24^+^CD38^hi^ Breg cell generation might also depend on the integration of other inflammatory signals, in particular the levels of CD40L expression, or the profile of cytokines produced over the course of the infection by contiguous or interacting cells. The finding that higher concentrations of IFN-α favor plasmablast generation might reflect a disease setting with chronic inflammation. For example, IFN-α overexposure might explain the lack of functional CD24^+^CD38^hi^ Breg cells, and bias toward anti-nuclear antibody production and more severe disease, in patients with SLE who have an “IFN gene signature.” In MS patients, IFN-β treatment can induce the development of thyroid autoimmunity (Frisullo et al., 2014) and failure to respond to treatment in patients with an existing “IFN gene signature” (Verweij and Vosslamber, 2013). IFN therapy in hepatitis C or chronic myelogenous leukemia (CML) patients can result in lupus-like symptoms (Tóthová, 2002, Wilson et al., 2002). Therefore, it is possible that the induction of autoimmune-like disease after type I IFN therapy in these cases might be due to the high concentrations of exogenous type I IFN favoring plasmablasts over CD24^+^CD38^hi^ Breg cell generation.

The analysis of CD24^+^CD38^hi^ B cells from SLE patients revealed an altered activation of STAT1 and STAT3 upon IFN-α stimulation, compared to healthy individuals. Among the cascade of events initiated by the IFN receptor engagement, phosphorylation of STAT1 induces transcription of pro-inflammatory genes, whereas phosphorylation of STAT3 indirectly suppresses pro-inflammatory gene expression and induces the transcription of IL-10 (Benkhart et al., 2000, Ivashkiv and Donlin, 2014, Luu et al., 2014). This supports our findings showing increased amounts of IL-6 and TNF-α and lower amounts of IL-10, after stimulation via pDCs in SLE CD24^+^CD38^hi^ B cells.

Our data suggest that the immune regulatory feedback between CD24^+^CD38^hi^ Breg cells and pDCs that is in place in healthy individuals is dysfunctional in SLE patients but that this feedback is normalized in patients who respond to rituximab. It is tempting to suggest that when sufficient CD24^+^CD38^hi^ Breg cells have been generated at a site of inflammation by IFN-α^+^ pDCs or after CD40-CD40L interactions with T cells, for instance, Breg cells can then prevent pDCs from producing excessive IFN-α that would otherwise drive chronic inflammation. A similar feedback mechanism was previously shown in neonatal mice, where B cells producing IL-10 control the inflammatory response mounted by pDCs after the systemic administration of a TLR-9 agonist (Zhang et al., 2007). However, in contrast to our results, this feature was restricted to neonatal B cells; TLR9 administration exacerbated inflammation in adult mice. Our data suggest that in adult humans, loss of feedback between CD24^+^CD38^hi^ Breg cells and pDCs correlates with loss of tolerance that was restored in patients responding to rituximab. Thus, in addition to simply depleting pathogenic B cells and restoring Breg cell number and function, as has been previously reported (Anolik et al., 2007, Bosma et al., 2012), rituximab might also normalize CD24^+^CD38^hi^ Breg cell-pDC interactions, supporting tolerance. Evaluating rituximab-treated patients in terms of their CD24^+^CD38^hi^ Breg cell frequency and pDC activation together reveals two groups of responders: responders that look similar to healthy controls and responders who look more similar to non-responders. It is possible that the responders whose CD24^+^CD38^hi^ Breg cell-pDC interactions do not appear to be normalized by rituximab treatment might be those who subsequently relapse to active disease.

Here, we report that pDCs control the differentiation of immature B cells into CD24^+^CD38^hi^ Breg cells or plasmablasts and that pDC-induced CD24^+^CD38^hi^ Breg cells might reciprocally regulate pDC-derived IFN-α production. We propose that this crosstalk is disrupted in SLE patients and that rituximab therapy might work, in part, by resetting the outcome of CD24^+^CD38^hi^ Breg cell-pDC interaction. Thus, modulation of the feedback loop between pDCs and CD24^+^CD38^hi^ Breg cells could potentially create new opportunities for immune-based therapies.

## Experimental Procedures

### Patients and Controls

Peripheral blood was obtained from healthy individuals or patients with SLE. Table S1 provides definition of disease activity and summarizes the characteristics of the SLE patients. Ethical approval was obtained from the Ethics Committee of UCLH-National Health Service Trust.

### Cell Isolation and Cultures

B cell subsets, CD4^+^CD25^−^ T cells, CD14^+^ monocytes, BDCA-3^+^ cDCs, and PBMCs depleted of pDCs were sorted on a FACSAria. CD19^+^ B cells or BCDA-2^+^CD123^+^ pDCs were isolated by magnetic-bead purification with EasySep enrichment kits (StemCell).

Isolated CD19^+^ B cells or FACS-sorted B cell subsets were cultured alone or with BDCA-2^+^CD123^+^ pDCs (unless otherwise stated) at 3:1 and stimulated with 1 μM of CpGC for 3 days. Cytokines were measured by intracellular staining. Further details about sample source, cell isolation procedures, B cell differentiation, and functional assays are included in Supplemental Experimental Procedures.

### Flow Cytometry

See Supplemental Experimental Procedures for the list of antibodies used. Cells were analyzed on a LSR II (BD Biosciences). Data were analyzed with FlowJo (Tree Star).

### Image Stream

PBMCs were stimulated with CpGC for 6 hr. The cells were stained for surface expression of CD19 and BDCA-2, fixed, permeabilized, and stained for intracellular IL-10. The sample was processed on an Amnis ImagetreamX Imaging Flow Cytometer (MERK-Millipore) with a 40× objective. Raw image files were acquired with INSPIRE software. A compensation matrix was then applied to the acquired data to correct for spectral overlap followed by data analysis by IDEAS 5.0 software. A combined Area to Aspect Ratio dot plot was used to identify doublets, and a gradient RMS for the Brightfield was used to exclude out of focus cells. Cells were gated as CD19^+^BDCA-2^+^ for pDC-B cell conjugate identification and assessed for the expression of IL-10.

### Quantification of Gene Expression by RT-PCR

Total RNA was extracted from B cells or pDCs using PicoPure RNA Isolation Kit (Life Technologies), according to manufacturer’s instructions. RNA was reverse-transcribed to cDNA with the iScript Reverse Transcription Supermix (BioRad), and gene expression was measured by quantitative RT-PCR. PCR primers used were as follows: actin (forward, 5′-AGA TGA CCC AGA TCA TGT TTG AG-3′; reverse, 5′-AGG TCC AGA CGC AGG ATG-3′), or *IFNA1*, *IRF7*, *IRF9*, *IL6*, *IL12A*, *IL12B*, *MX1*, *MCL1*, and *TLR9* QuantiTect primers (QIAGEN). The relative expression level of specific transcripts was normalized with respect to the internal standard, β-actin. Relative expression was calculated against healthy control via the ΔΔC_T_ method.

### Cell Signaling

PBMCs were surface stained with CD19, CD24, and CD38 antibodies in ice-cold PBS for 30 min, then rested for 1 hr in RPMI medium. Cells were stimulated by incubating with 10,000 U/mL exogenous IFN-α (PBL) for 1 or 5 min and then fixed with formaldehyde for 10 min at 37°C, washed with ice-cold PBS, and permeabilized with 90% ice-cold methanol for 20 min on ice. tSTAT1-PE, tSTAT3-AF647, pSTAT1-AF647, and pSTAT3-PE (BD Phosflow) were added to cells, for 1 hr at 37°C.

### Statistical Analysis

Statistical analysis was performed with the Prism Software (GraphPad) by paired or unpaired t test or one-way or two-way ANOVA as specified, with Bonferroni correction for multiple comparisons. Correlations were assessed with Pearson’s correlation coefficient. A p value of ≤ 0.05 was considered significant. ns, not significant; ^∗^p < 0.05, ^∗∗^p < 0.01, ^∗∗∗^p < 0.001.

## Author Contributions

M.M. performed the experiments. C.M. and M.M. designed the experiments. C.M., M.M., and P.A.B. wrote the manuscript. D.A.I. provided SLE samples and clinical expertise.

## Supplemental Information

Document S1. Figures S1–S7, Table S1, and Supplemental Experimental Procedures

Document S2. Article plus Supplemental Information

## References

[bib1] Anolik J.H., Barnard J., Owen T., Zheng B., Kemshetti S., Looney R.J., Sanz I. (2007). Delayed memory B cell recovery in peripheral blood and lymphoid tissue in systemic lupus erythematosus after B cell depletion therapy. Arthritis Rheum..

[bib2] Baccala R., Gonzalez-Quintial R., Schreiber R.D., Lawson B.R., Kono D.H., Theofilopoulos A.N. (2012). Anti-IFN-α/β receptor antibody treatment ameliorates disease in lupus-predisposed mice. J. Immunol..

[bib3] Baccala R., Gonzalez-Quintial R., Blasius A.L., Rimann I., Ozato K., Kono D.H., Beutler B., Theofilopoulos A.N. (2013). Essential requirement for IRF8 and SLC15A4 implicates plasmacytoid dendritic cells in the pathogenesis of lupus. Proc. Natl. Acad. Sci. USA.

[bib4] Benkhart E.M., Siedlar M., Wedel A., Werner T., Ziegler-Heitbrock H.W. (2000). Role of Stat3 in lipopolysaccharide-induced IL-10 gene expression. J. Immunol..

[bib5] Bennett L., Palucka A.K., Arce E., Cantrell V., Borvak J., Banchereau J., Pascual V. (2003). Interferon and granulopoiesis signatures in systemic lupus erythematosus blood. J. Exp. Med..

[bib6] Blair P.A., Noreña L.Y., Flores-Borja F., Rawlings D.J., Isenberg D.A., Ehrenstein M.R., Mauri C. (2010). CD19(+)CD24(hi)CD38(hi) B cells exhibit regulatory capacity in healthy individuals but are functionally impaired in systemic Lupus Erythematosus patients. Immunity.

[bib7] Blanco P., Palucka A.K., Gill M., Pascual V., Banchereau J. (2001). Induction of dendritic cell differentiation by IFN-alpha in systemic lupus erythematosus. Science.

[bib8] Bosma A., Abdel-Gadir A., Isenberg D.A., Jury E.C., Mauri C. (2012). Lipid-antigen presentation by CD1d(+) B cells is essential for the maintenance of invariant natural killer T cells. Immunity.

[bib9] Cederblad B., Blomberg S., Vallin H., Perers A., Alm G.V., Rönnblom L. (1998). Patients with systemic lupus erythematosus have reduced numbers of circulating natural interferon-alpha- producing cells. J. Autoimmun..

[bib10] Duramad O., Fearon K.L., Chan J.H., Kanzler H., Marshall J.D., Coffman R.L., Barrat F.J. (2003). IL-10 regulates plasmacytoid dendritic cell response to CpG-containing immunostimulatory sequences. Blood.

[bib11] Feng X., Wu H., Grossman J.M., Hanvivadhanakul P., FitzGerald J.D., Park G.S., Dong X., Chen W., Kim M.H., Weng H.H. (2006). Association of increased interferon-inducible gene expression with disease activity and lupus nephritis in patients with systemic lupus erythematosus. Arthritis Rheum..

[bib12] Flores-Borja F., Bosma A., Ng D., Reddy V., Ehrenstein M.R., Isenberg D.A., Mauri C. (2013). CD19+CD24hiCD38hi B cells maintain regulatory T cells while limiting TH1 and TH17 differentiation. Sci. Transl. Med..

[bib13] Francisco L.M., Salinas V.H., Brown K.E., Vanguri V.K., Freeman G.J., Kuchroo V.K., Sharpe A.H. (2009). PD-L1 regulates the development, maintenance, and function of induced regulatory T cells. J. Exp. Med..

[bib14] Frisullo G., Calabrese M., Tortorella C., Paolicelli D., Ragonese P., Annovazzi P., Radaelli M., Malucchi S., Gallo A., Tomassini V. (2014). Thyroid autoimmunity and dysfunction in multiple sclerosis patients during long-term treatment with interferon beta or glatiramer acetate: an Italian multicenter study. Mult. Scler..

[bib15] Garcia-Romo G.S., Caielli S., Vega B., Connolly J., Allantaz F., Xu Z., Punaro M., Baisch J., Guiducci C., Coffman R.L. (2011). Netting neutrophils are major inducers of type I IFN production in pediatric systemic lupus erythematosus. Sci. Transl. Med..

[bib16] Georg P., Bekeredjian-Ding I. (2012). Plasmacytoid dendritic cells control B cell-derived IL-10 production. Autoimmunity.

[bib17] Gilliet M., Cao W., Liu Y.J. (2008). Plasmacytoid dendritic cells: sensing nucleic acids in viral infection and autoimmune diseases. Nat. Rev. Immunol..

[bib18] Hartmann G., Battiany J., Poeck H., Wagner M., Kerkmann M., Lubenow N., Rothenfusser S., Endres S. (2003). Rational design of new CpG oligonucleotides that combine B cell activation with high IFN-alpha induction in plasmacytoid dendritic cells. Eur. J. Immunol..

[bib19] Hoffmann H.H., Schneider W.M., Rice C.M. (2015). Interferons and viruses: an evolutionary arms race of molecular interactions. Trends Immunol..

[bib20] Honda K., Yanai H., Negishi H., Asagiri M., Sato M., Mizutani T., Shimada N., Ohba Y., Takaoka A., Yoshida N., Taniguchi T. (2005). IRF-7 is the master regulator of type-I interferon-dependent immune responses. Nature.

[bib21] Ito T., Yang M., Wang Y.H., Lande R., Gregorio J., Perng O.A., Qin X.F., Liu Y.J., Gilliet M. (2007). Plasmacytoid dendritic cells prime IL-10-producing T regulatory cells by inducible costimulator ligand. J. Exp. Med..

[bib22] Ivashkiv L.B., Donlin L.T. (2014). Regulation of type I interferon responses. Nat. Rev. Immunol..

[bib23] Jego G., Palucka A.K., Blanck J.P., Chalouni C., Pascual V., Banchereau J. (2003). Plasmacytoid dendritic cells induce plasma cell differentiation through type I interferon and interleukin 6. Immunity.

[bib24] Kerkmann M., Rothenfusser S., Hornung V., Towarowski A., Wagner M., Sarris A., Giese T., Endres S., Hartmann G. (2003). Activation with CpG-A and CpG-B oligonucleotides reveals two distinct regulatory pathways of type I IFN synthesis in human plasmacytoid dendritic cells. J. Immunol..

[bib25] Lande R., Ganguly D., Facchinetti V., Frasca L., Conrad C., Gregorio J., Meller S., Chamilos G., Sebasigari R., Riccieri V. (2011). Neutrophils activate plasmacytoid dendritic cells by releasing self-DNA-peptide complexes in systemic lupus erythematosus. Sci. Transl. Med..

[bib26] Leandro M.J., Cambridge G., Ehrenstein M.R., Edwards J.C. (2006). Reconstitution of peripheral blood B cells after depletion with rituximab in patients with rheumatoid arthritis. Arthritis Rheum..

[bib27] Lechmann M., Berchtold S., Hauber J., Steinkasserer A. (2002). CD83 on dendritic cells: more than just a marker for maturation. Trends Immunol..

[bib28] Li H., Fu Y.-X., Wu Q., Zhou Y., Crossman D.K., Yang P., Li J., Lao B., Morel L.M., Kabarowski J.H. (2015). Interferon-induced mechanosensing defects impede apoptotic cell clearance in lupus. J. Clin. Invest..

[bib29] Luu K., Greenhill C.J., Majoros A., Decker T., Jenkins B.J., Mansell A. (2014). STAT1 plays a role in TLR signal transduction and inflammatory responses. Immunol. Cell Biol..

[bib30] Malaspina A., Moir S., Ho J., Wang W., Howell M.L., O’Shea M.A., Roby G.A., Rehm C.A., Mican J.M., Chun T.W., Fauci A.S. (2006). Appearance of immature/transitional B cells in HIV-infected individuals with advanced disease: correlation with increased IL-7. Proc. Natl. Acad. Sci. USA.

[bib31] Matsumoto M., Baba A., Yokota T., Nishikawa H., Ohkawa Y., Kayama H., Kallies A., Nutt S.L., Sakaguchi S., Takeda K. (2014). Interleukin-10-producing plasmablasts exert regulatory function in autoimmune inflammation. Immunity.

[bib32] Mauri C., Bosma A. (2012). Immune regulatory function of B cells. Annu. Rev. Immunol..

[bib33] Mauri C., Nistala K. (2014). Interleukin-35 takes the ‘B’ line. Nat. Med..

[bib34] McKenna K., Beignon A.S., Bhardwaj N. (2005). Plasmacytoid dendritic cells: linking innate and adaptive immunity. J. Virol..

[bib35] Moseman E.A., Liang X., Dawson A.J., Panoskaltsis-Mortari A., Krieg A.M., Liu Y.J., Blazar B.R., Chen W. (2004). Human plasmacytoid dendritic cells activated by CpG oligodeoxynucleotides induce the generation of CD4+CD25+ regulatory T cells. J. Immunol..

[bib36] Obermoser G., Pascual V. (2010). The interferon-alpha signature of systemic lupus erythematosus. Lupus.

[bib37] Pascual V., Farkas L., Banchereau J. (2006). Systemic lupus erythematosus: all roads lead to type I interferons. Curr. Opin. Immunol..

[bib38] Poeck H., Wagner M., Battiany J., Rothenfusser S., Wellisch D., Hornung V., Jahrsdorfer B., Giese T., Endres S., Hartmann G. (2004). Plasmacytoid dendritic cells, antigen, and CpG-C license human B cells for plasma cell differentiation and immunoglobulin production in the absence of T-cell help. Blood.

[bib39] Reizis B., Bunin A., Ghosh H.S., Lewis K.L., Sisirak V. (2011). Plasmacytoid dendritic cells: recent progress and open questions. Annu. Rev. Immunol..

[bib40] Rivero S.J., Díaz-Jouanen E., Alarcón-Segovia D. (1978). Lymphopenia in systemic lupus erythematosus. Clinical, diagnostic, and prognostic significance. Arthritis Rheum..

[bib41] Rönnblom L., Eloranta M.L. (2013). The interferon signature in autoimmune diseases. Curr. Opin. Rheumatol..

[bib42] Rosser E.C., Blair P.A., Mauri C. (2014). Cellular targets of regulatory B cell-mediated suppression. Mol. Immunol..

[bib43] Rowland S.L., Riggs J.M., Gilfillan S., Bugatti M., Vermi W., Kolbeck R., Unanue E.R., Sanjuan M.A., Colonna M. (2014). Early, transient depletion of plasmacytoid dendritic cells ameliorates autoimmunity in a lupus model. J. Exp. Med..

[bib44] Schubert R.D., Hu Y., Kumar G., Szeto S., Abraham P., Winderl J., Guthridge J.M., Pardo G., Dunn J., Steinman L., Axtell R.C. (2015). IFN-β treatment requires B cells for efficacy in neuroautoimmunity. J. Immunol..

[bib45] Shibuya H., Hirohata S. (2005). Differential effects of IFN-alpha on the expression of various TH2 cytokines in human CD4+ T cells. J. Allergy Clin. Immunol..

[bib46] Siegal F.P., Kadowaki N., Shodell M., Fitzgerald-Bocarsly P.A., Shah K., Ho S., Antonenko S., Liu Y.J. (1999). The nature of the principal type 1 interferon-producing cells in human blood. Science.

[bib47] Siewe B., Stapleton J.T., Martinson J., Keshavarzian A., Kazmi N., Demarais P.M., French A.L., Landay A. (2013). Regulatory B cell frequency correlates with markers of HIV disease progression and attenuates anti-HIV CD8^+^ T cell function in vitro. J. Leukoc. Biol..

[bib48] Sisirak V., Ganguly D., Lewis K.L., Couillault C., Tanaka L., Bolland S., D’Agati V., Elkon K.B., Reizis B. (2014). Genetic evidence for the role of plasmacytoid dendritic cells in systemic lupus erythematosus. J. Exp. Med..

[bib49] Swiecki M., Colonna M. (2015). The multifaceted biology of plasmacytoid dendritic cells. Nat. Rev. Immunol..

[bib50] Thibault D.L., Chu A.D., Graham K.L., Balboni I., Lee L.Y., Kohlmoos C., Landrigan A., Higgins J.P., Tibshirani R., Utz P.J. (2008). IRF9 and STAT1 are required for IgG autoantibody production and B cell expression of TLR7 in mice. J. Clin. Invest..

[bib51] Tóthová E. (2002). [New views on the treatment of chronic myelocytic leukemia. Literature review]. Vnitr. Lek..

[bib52] Verweij C.L., Vosslamber S. (2013). Relevance of the type I interferon signature in multiple sclerosis towards a personalized medicine approach for interferon-beta therapy. Discov. Med..

[bib53] Wilson L.E., Widman D., Dikman S.H., Gorevic P.D. (2002). Autoimmune disease complicating antiviral therapy for hepatitis C virus infection. Semin. Arthritis Rheum..

[bib54] Yoshizaki A., Miyagaki T., DiLillo D.J., Matsushita T., Horikawa M., Kountikov E.I., Spolski R., Poe J.C., Leonard W.J., Tedder T.F. (2012). Regulatory B cells control T-cell autoimmunity through IL-21-dependent cognate interactions. Nature.

[bib55] Zhang X., Deriaud E., Jiao X., Braun D., Leclerc C., Lo-Man R. (2007). Type I interferons protect neonates from acute inflammation through interleukin 10-producing B cells. J. Exp. Med..

