# Supplementary material for: A Regulatory Feedback between Plasmacytoid Dendritic Cells and Regulatory B Cells Is Aberrant in Systemic Lupus Erythematosus
Source: Immunity. 2016 Mar 15;44(3):683–97. doi: 10.1016/j.immuni.2016.02.012 (PMC4803914; doi:10.1016/j.immuni.2016.02.012)
Supplement: Document S1. Figures S1–S7, Table S1, and Supplemental Experimental Procedures [file mmc1.pdf]

Immunity, Volume *44*

Supplemental Information

A Regulatory Feedback between Plasmacytoid Dendritic Cells and Regulatory B Cells Is Aberrant in Systemic Lupus Erythematosus

Madhvi Menon, Paul A. Blair, David A. Isenberg, and Claudia Mauri

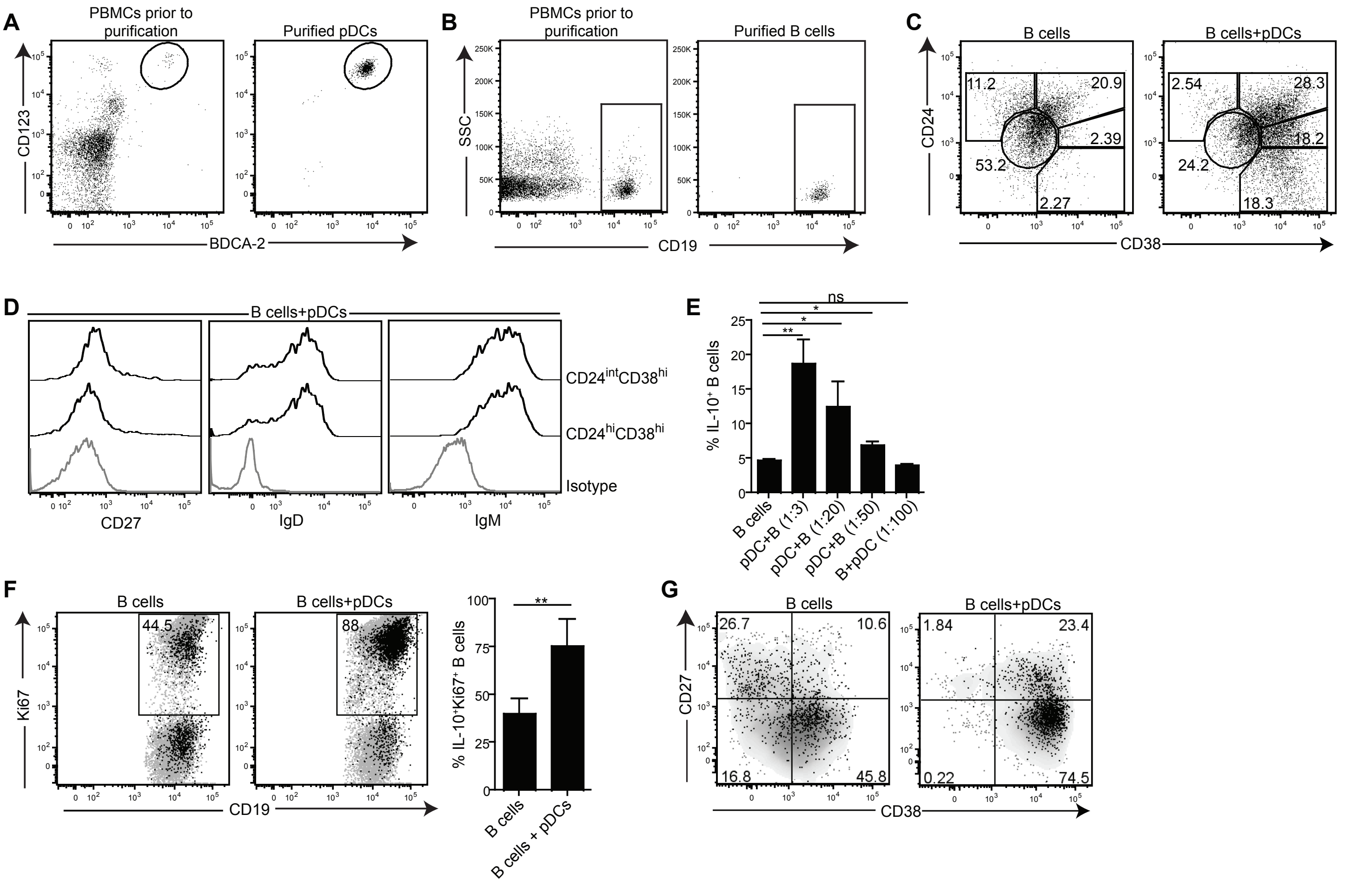

Figure S1, related to Figure 1. pDCs expand IL-10$^+$ B cells with a CD24$^+$CD38^{hi}CD27$^-$IgD^{hi}IgM^{hi} phenotype. (A) Representative FACS plots depict the BDCA-2$^+$CD123$^+$ pDC purity before and after isolation using a pDC enrichment kit. (B) Representative FACS plots depict the CD19$^+$B cell purity before and after isolation using a B cell enrichment kit. (C) Represntative FACS plots showing CD24^{hi}CD38^{hi}, CD24^{int}CD38^{hi}, CD24$^+$CD38$^-$, CD24^{int}CD38^{int} and CD24$^-$CD38^{hi} B cell subsets following 72 hr culture of B cells with CpGC or CpGC and autologous pDCs. (D) Representative histograms displaying CD27, IgD and IgM expression on CD24$^+$CD38^{hi} and CD24$^-$CD38^{hi} B cell subsets following culture with autologous pDCs and CpGC. (E) Bar chart depicting the frequency of IL-10$^+$B cells in co-cultures of B cells with pDCs at different ratios (3:1, 20:1, 50:1 and 100:1 B:pDC) stimulated with CpGC; mean ± s.e.m. in 4 healthy donors. (F) Representative FACS plot showing IL-10$^+$B cells (black dots) overlaid on a plot depicting Ki67 expression by CD19$^+$B cells (gray dot plot) after culture of B cells with CpGC alone, or with CpGC and pDCs for 72 hrs. Bar chart showing the frequency of IL-10$^+$Ki67$^+$B cells as mean ± s.e.m. in healthy donors (n=4). (G) Representative flow cytometry plots overlaying IL-10$^+$ B cells (black dots) on total B cells (grey density plot) following B cell and pDC co-culture with CpGC; gated on CD27 and CD38 expression of B cells. Data are representative of three independent experiments (C-D, G). ns, not significant; *$p<0.05$, **$p<0.01$; paired t test (F), one-way ANOVA (E).

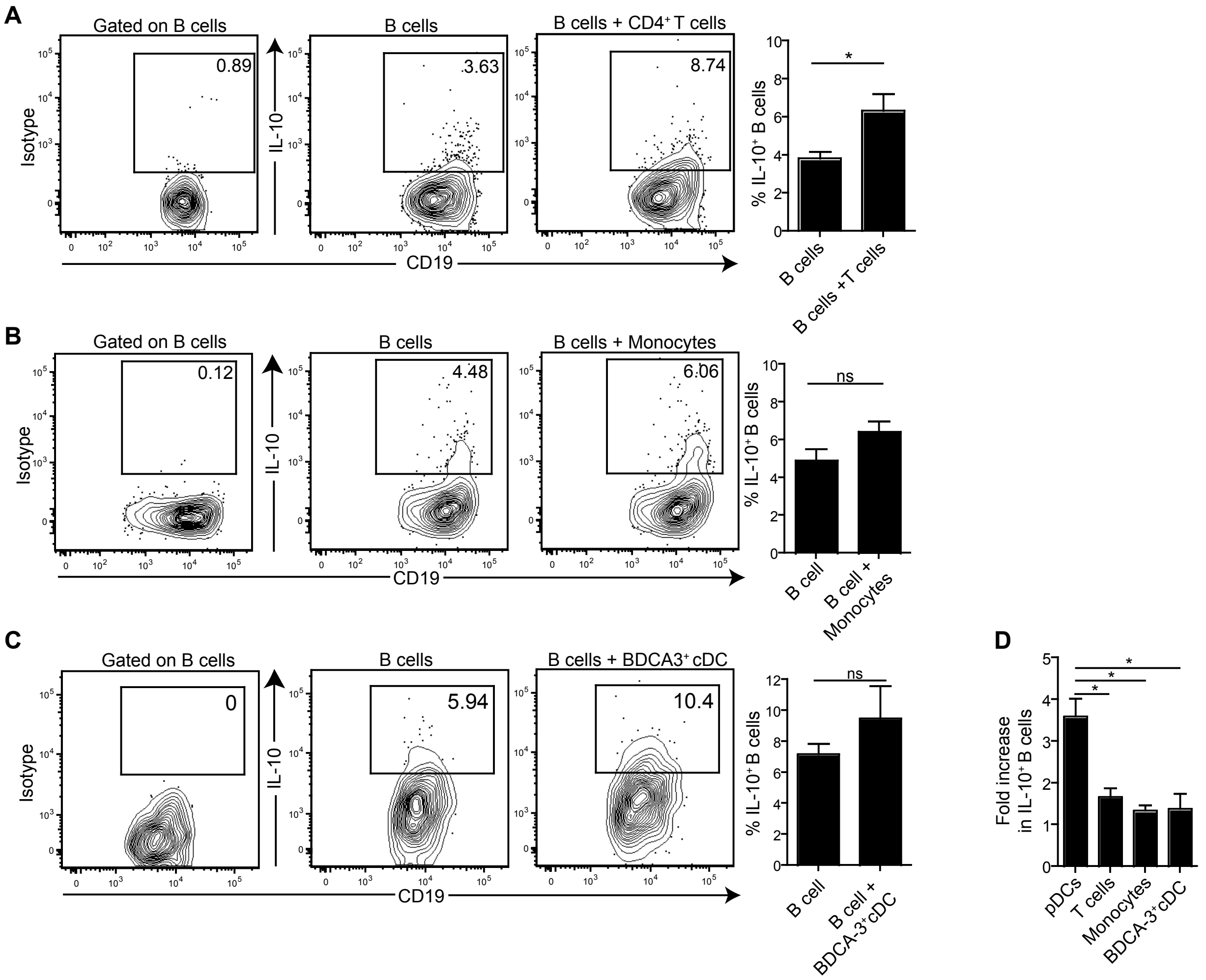

Figure S2, related to Figure 1. pDCs are more efficient than CD4$^+$ T cells, monocytes and BDCA-3$^+$ cDCs in the expansion of IL-10-producing B cells.

(A) Representative plots and bar graph displaying the frequency of IL-10$^+$ B cells after culture of B cells alone or with CD4$^+$ T cells, stimulated with CpGC and plate-bound anti-CD3 for 72 hrs (n=3 healthy donors, mean ± s.e.m.). (B) Representative plots and bar graph displaying the frequency of IL-10$^+$ B cells after culture of B cells with or without monocytes, stimulated with CpGC and R848 for 72 hrs in n=3 healthy donors, mean ± s.e.m. (C) Representative plots and bar graph displaying the frequency of IL-10$^+$ B cells after culture of B cells alone or with BDCA-3$^+$ cDCs, stimulated with CpGC and Poly I:C for 72 hrs in n=3 healthy donors, mean ± s.e.m. (D) Bar chart displays the fold increase in IL-10$^+$B cells on culture of B cells with pDCs, CD4$^+$ T cells, monocytes or BDCA-3$^+$ cDCs. Data are representative of two independent experiments (A-B). ns, not significant; *p<0.05, **p<0.01; paired t test (A-C), one-way ANOVA (D).

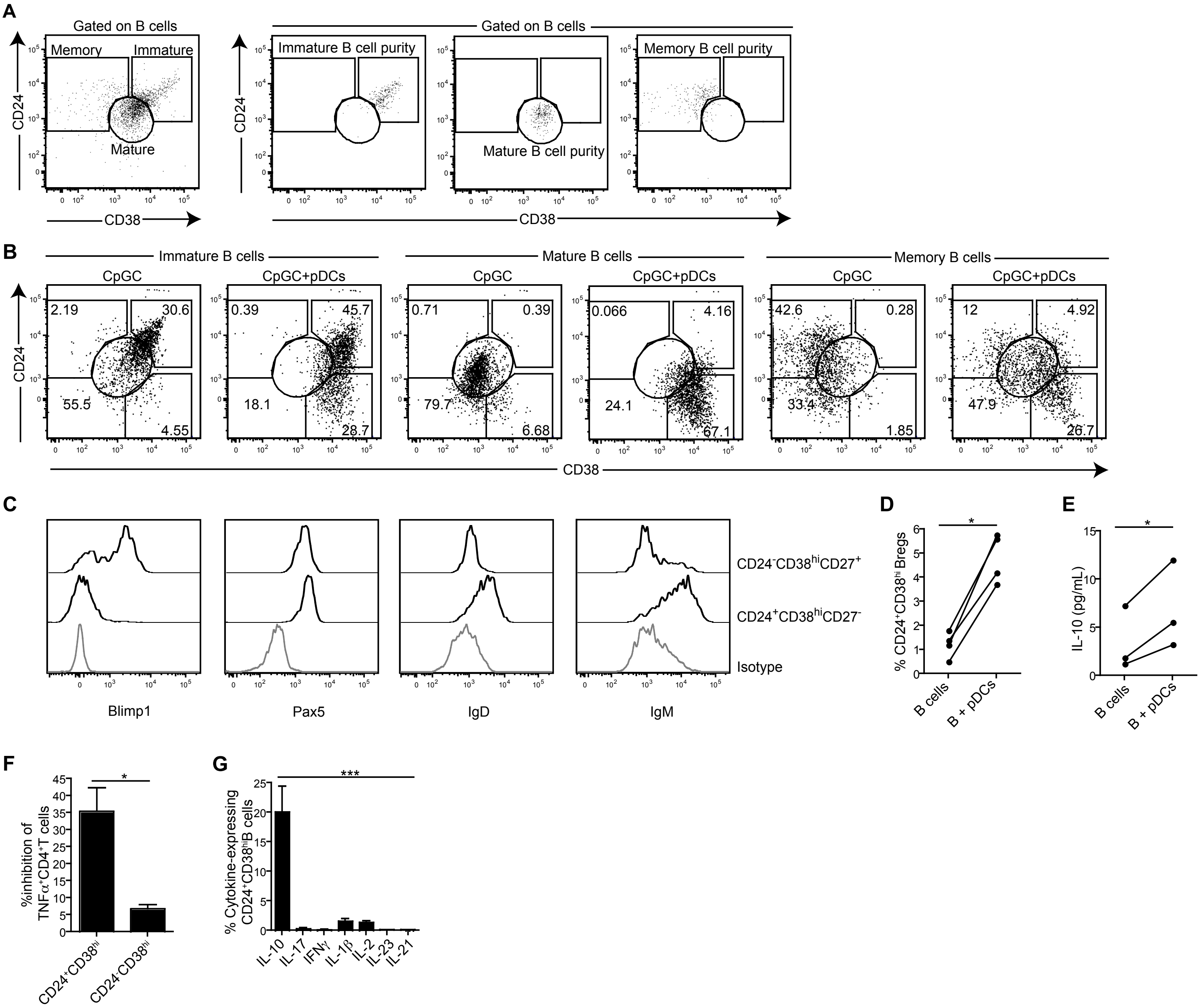

Figure S3, related to Figure 2. TLR-activated pDCs expand $CD24^{hi}CD38^{hi}$Bregs and plasmablasts.
(A) Representative FACS plots depict the $CD19^{+}$B cell purity before and after isolation using a B cell enrichment kit. (B) Representative FACS plots showing frequency of $CD24^{+}CD38^{hi}$, $CD24^{int}CD38^{int}$, $CD24^{+}CD38^{-}$ and $CD24^{-}$ $CD38^{hi}$ B cell subsets, after FACS-sorted immature, mature and memory B cells were cultured with CpGC and pDCs or CpGC alone. (C) Representative histograms displaying the expression of Blimp1, Pax5, IgD and IgM of immature B cell-derived $CD24^{+}CD38^{hi}CD27^{-}$ and $CD24^{-}CD38^{hi}CD27^{+}$ B cell subsets. (D) Cumulative data displaying the frequency of $CD24^{+}CD38^{hi}$Bregs after co-culture of B cells with pDCs and human CMV lysates for 72 hours (n=4). (E) Cumulative data shows the IL-10 measured in the culture supernatants in C (n=3). (F) Bar chart showing the percentage inhibition of $TNF\alpha^{+}CD4^{+}$T cells by immature B cell-derived $CD24^{+}CD38^{hi}$ and $CD24^{-}CD38^{hi}$B cell subsets, from n=3 healthy individuals as mean ± s.e.m. *p<0.05; unpaired t test. (G) Bar chart showing the expression of cytokines by $CD24^{+}CD38^{hi}$B cells upon stimulation with CpGC and pDCs for 72 hours (n=4 healthy donors, mean ± s.e.m.). ***p<0.001, one-way ANOVA. Data are representative of three independent experiments (B-E). *p<0.05; paired t test.

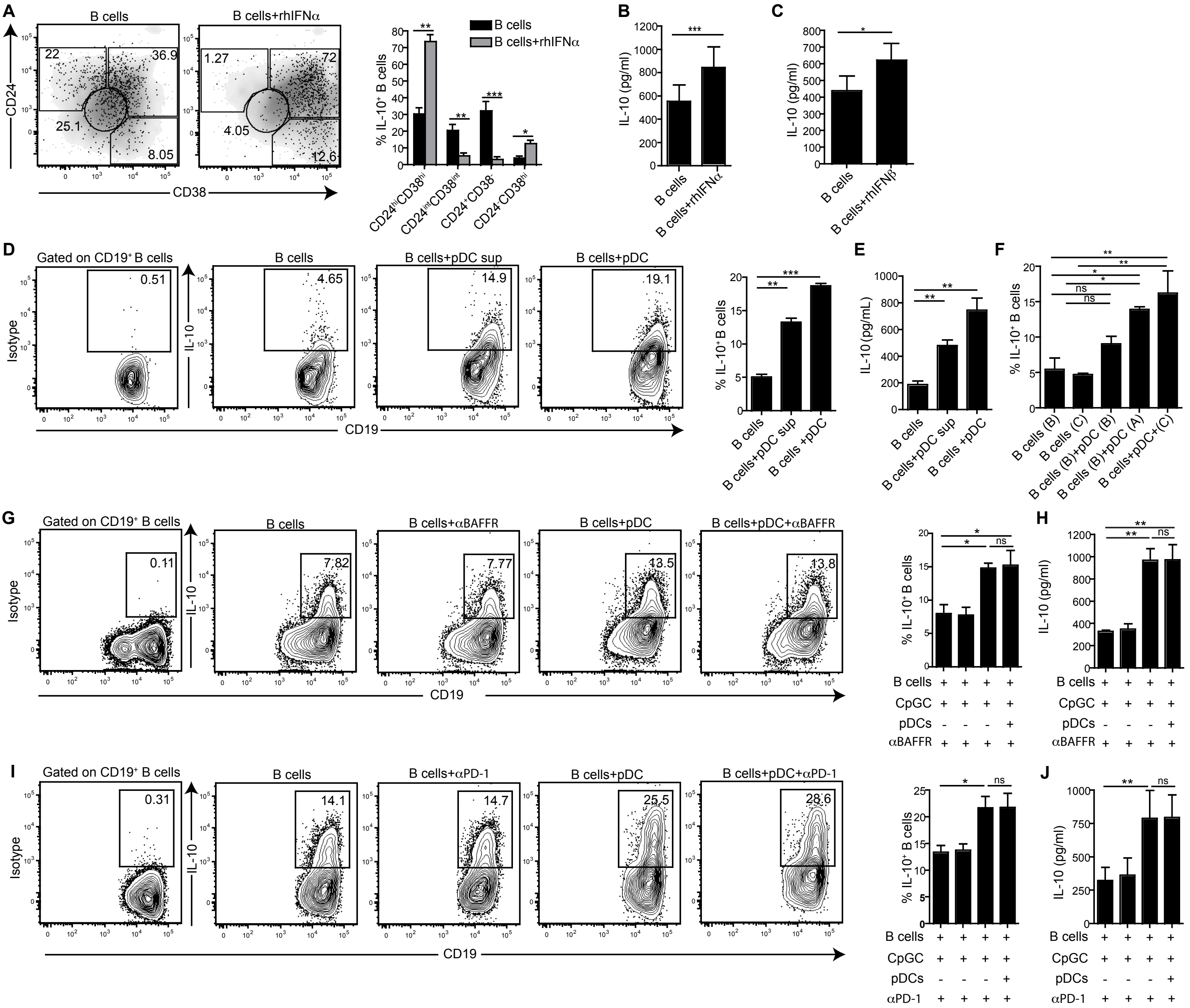

Figure S4, related to Figure 3. pDCs expand Bregs partially via IFNα or IFNβ, but independent of BAFF and PD1-PDL1 interaction.

(A) Representative FACS plot showing IL-10+ B cells (black dots) overlaid on a plot depicting CD24 and CD38 expression (gray density plot), after CD19+B cells were cultured with CpGC alone, or CpGC and exogenous IFNα (1000 U/ml) for 72 hrs. Bar chart showing the frequency of IL-10+ B cells within the B cell subsets as mean ± s.e.m. in n=7 healthy individuals. (B) Bar chart showing IL-10 concentration measured by ELISA in the supernatants collected from A. (C) Bar chart showing IL-10 in the supernatants collected from B cells cultured with CpGC and 1000U/ml IFNβ (mean ± s.e.m. of n=5). (D) Representative FACS plots and cumulative data displaying the frequency of IL-10+ B cells from healthy individuals (n=4, mean ± s.e.m.), after 72 hr culture of B cells with pDCs and CpGC, or supernatants from CpGC-stimulated pDCs. (E) Bar chart displays IL-10 concentrations measured by ELISA in the supernatants collected in D. (F) Bar chart showing the frequency of IL-10+ B cells from co-cultures with CpGB (B)-stimulated B cells and either CpGA (A) or CpGB (B)-stimulated pDCs, or pDCs and B cells stimulated with CpGC (C) (mean ± s.e.m. of n=3). (G-J) Representative FACS plots and bar charts showing (G,I) IL-10 expression by flow cytometry, or (H,J) IL-10 concentration in supernatatnts by ELISA for CD19+ B cells cultured alone or in the presence of pDCs and indicated blocking antibodies, BAFFR mAb (10µg/ml) in (G,H) or PD-1 mAb (10µg/ml) in (I,J). (n=4, mean ± s.e.m.).
ns, not significant; *p<0.05, **p<0.01, ***p<0.001; two-way ANOVA (A), Paired t test (B,C), one-way ANOVA (D-I).

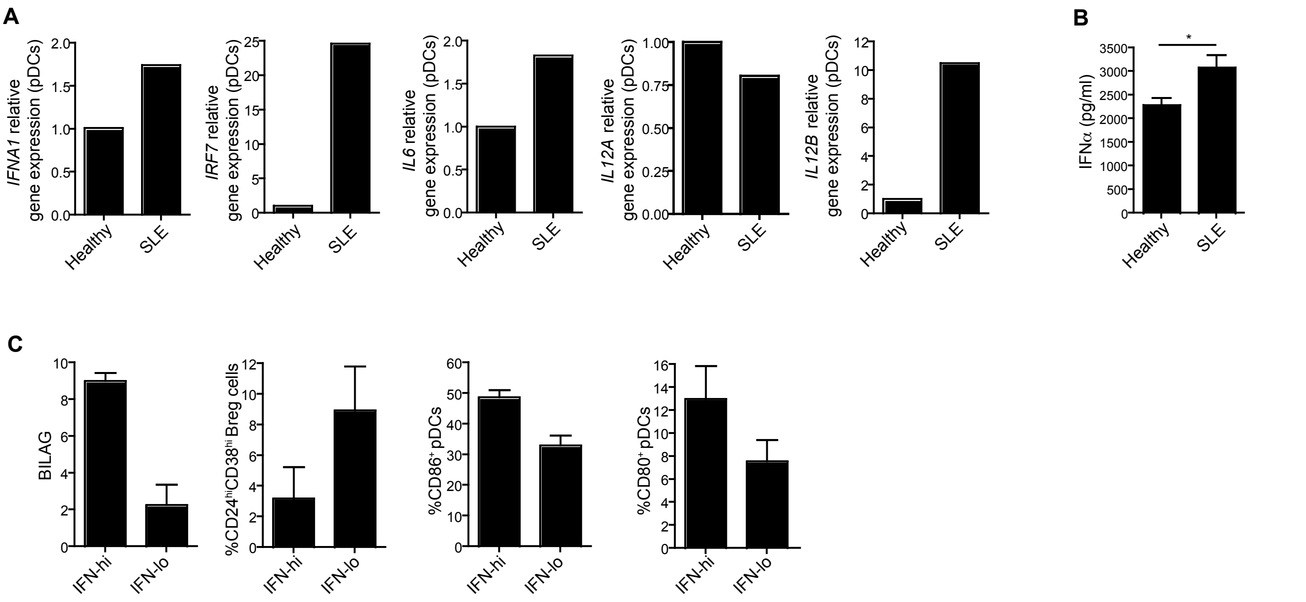

Figure S5, related to Figure 4. SLE patients with elevated IFN-I signature display a more active disease, reduced CD24^{hi}CD38^{hi} Breg cells and increased pDC activation.

(A) Bar charts showing the expression of genes *IFNA1*, *IRF7*, *IL6*, *IL12A* and *IL12B* on pooled pDCs from healthy individuals (n=4) and SLE patients (n=4). (B) Bar chart showing the IFNα concentration in the supernatants of CpGA-stimulated PBMC from n=20 healthy individuals and n=18 SLE patients. *$p<0.05$; unpaired t test. (C) SLE patients were grouped based on the gene expression of IRF9 as IFN-hi (IRF9 expression>4) and IFN-lo (IRF9 expression<4). Bar graphs showing the patient BILAG, frequency of CD24^{hi}CD38^{hi} Breg cells, frequency of CD86$^+$ pDCs and CD80$^+$ pDCs in the different groups.

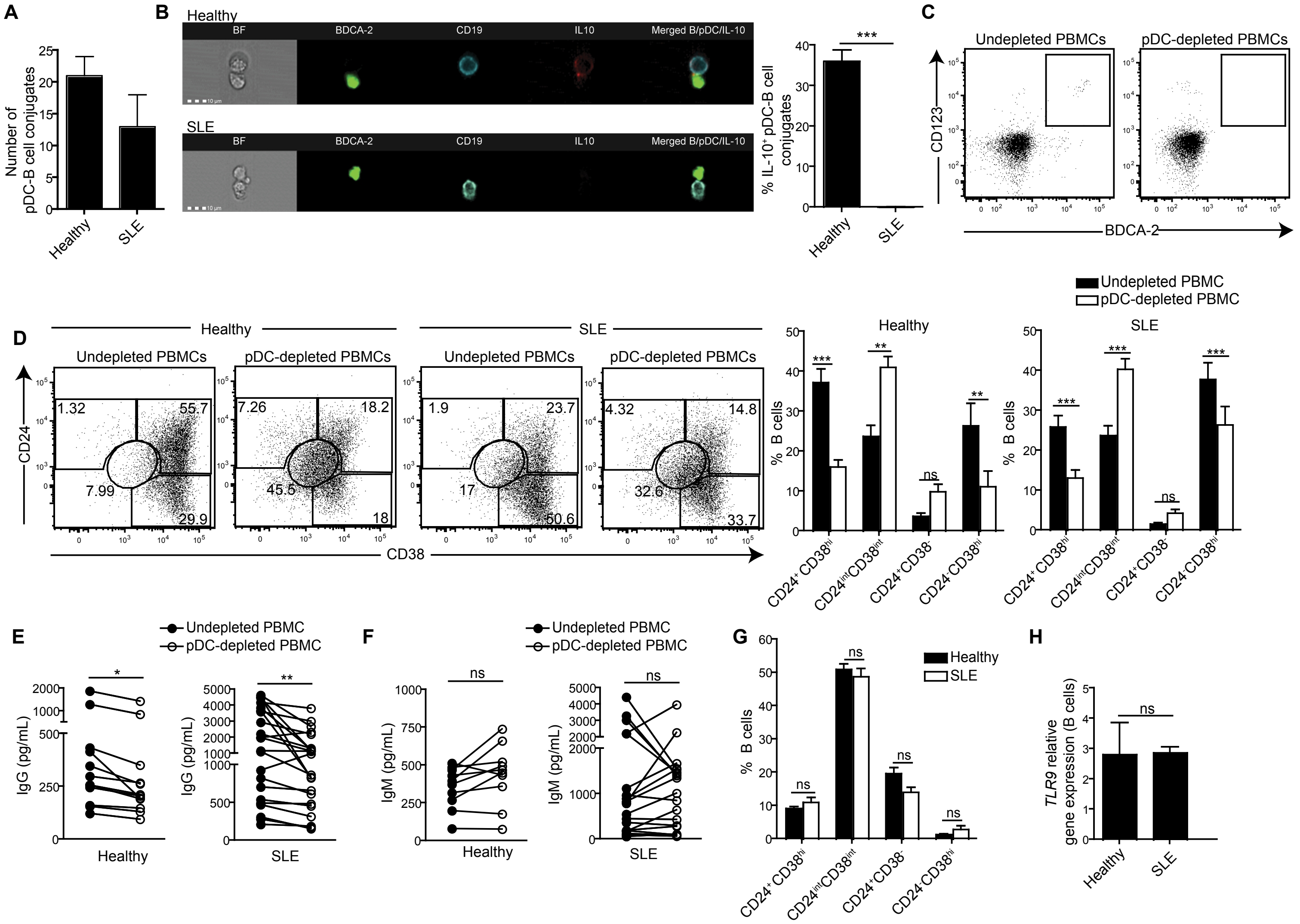

Figure S6, related to Figure 5. pDCs interacting with B cells promote plasmablast differentiation and IgG production.

(A) Bar chart showing the number of pDC-B cell conjugates using ImageStream analysis in healthy individuals (n=3) and SLE patients (n=3) after stimulation of PBMCs with CpGC for 6 hrs, as mean ± s.e.m. (B) Representative Imagestream ISX showing conjugate formation between $CD19^{+}$B cells (blue) and BDCA-2^{+}pDCs (green), with the expression of IL-10 on B cells (red). Bar shart showing the frequency of IL-10+ pDC-B cell conjugates in healthy individuals (n=3) and SLE patients (n=3), expressed as mean ± s.e.m. (C) Flow cytometry plots showing the pDC purity in the undepleted and pDC-depleted PBMCs. (D-F) pDC-depleted or undepleted PBMCs from healthy individuals and SLE patients were cultured with CpGC for 3 or 10 days. (D) Representative FACS plots displaying the frequency of $CD24^{hi}CD38^{hi}$, $CD24^{int}CD38^{int}$, $CD24^{+}CD38^{-}$ and CD24-$CD38^{hi}$B cell subsets after 3 days. Bar charts showing the frequency of B cell subsets in healthy individuals (n=11) and SLE patients (n=24) as mean ± s.e.m. (E and F) Cumulative data showing (E) IgG in n=14 healthy and n=22 SLE, and (F) IgM in n=12 healthy and n=18 SLE measured in the culture supernatants by ELISA after 10 days. ns, not significant; *p<0.05, ** p<0.01, ***p<0.001; two-way ANOVA (C,D), paired t test (E,F). (G) Bar chart displaying the frequency of $CD24^{+}CD38^{hi}$, $CD24^{int}CD38^{int}$, $CD24^{+}CD38^{-}$ and $CD24^{-}CD38^{hi}$B cell subsets *ex vivo* in healthy individuals (n=22) and SLE patients (n=19) as mean ± s.e.m. (H) Bar chart showing the gene expression of *TLR9* on B cells from healthy individuals (n=3) and SLE patients (n=3). ns, not significant; unpaired t test.

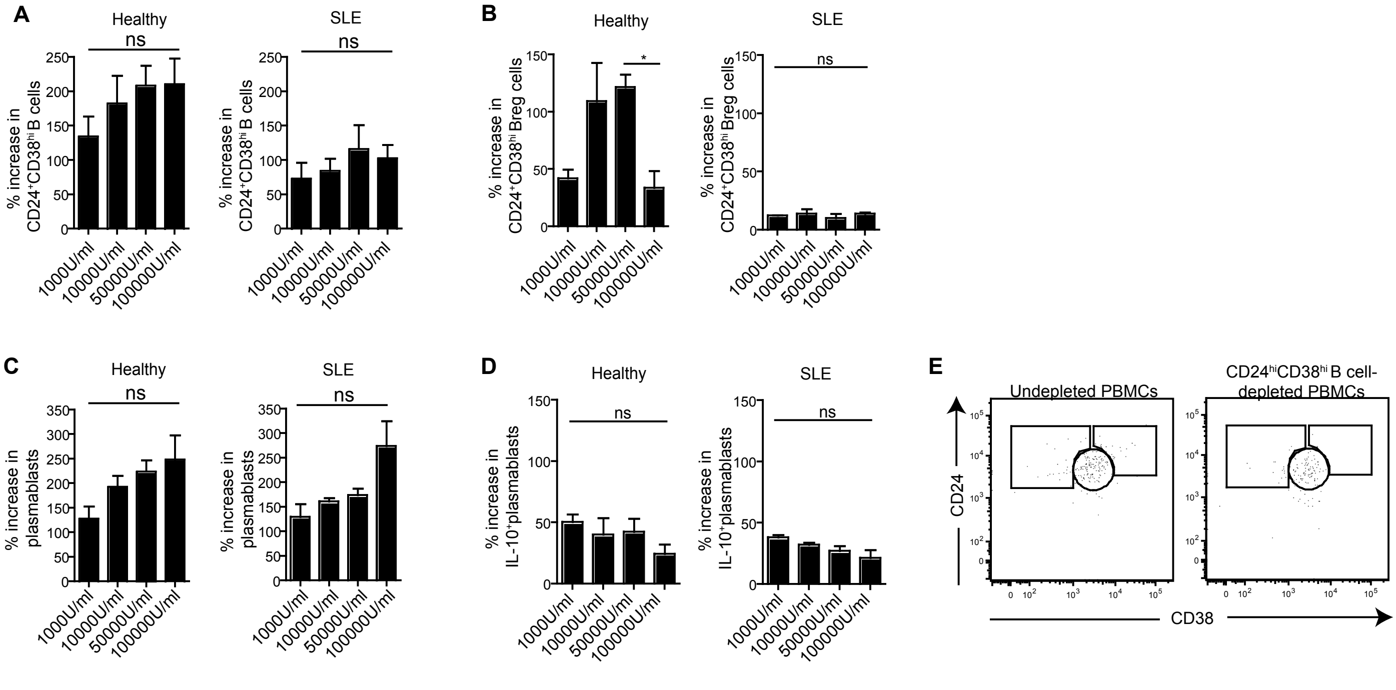

Figure S7, related to Figures 5 and 6. IFNα promotes B cell differentiation into plasmablasts in a dose-dependent manner.

(A-D) Bar chart showing the percentage increase in (A) CD24$^+$CD38^{hi}B cells, (B) CD24$^+$CD38^{hi} Breg cells, (C) plasmablasts and (D) IL-10$^+$ plasmablasts on stimulation of healthy or SLE B cells (n=4) with CpGC and 1000 U/ml, 10,000 U/ml, 50,000 U/ml, or 100,000 U/ml of IFNα, compared to stimulation of B cells with CpGC alone. Data are expressed as mean ± s.e.m. ns, not significant, *p<0.05; one-way ANOVA. (E) Flow cytometry plots showing the B cell subsets in undepleted and CD24^{hi}CD38^{hi}B cell-depleted PBMCs.

Supplementary Table 1, related to Figures 4, 5, 6 and 7. Summary of SLE patient clinical data, with or without rituximab treatment

	Healthy (n=88)	SLE (n=116)	Responder (n=59)	Non-responder (n=42)
Age (Range)	36.1 (20-58)	43.3 (21-79)	40.91 (26-65)	39.75 (24-64)
Sex (female: male)	(70:18)	(112:4)	(56:3)	(40:2)
Sex (% female: male)	(79.54: 20.45)	(96.5:3.5)	(94.9:5.1)	(95.2/4.8)
Ethnicity (C/A/AC/O)	(71/9/4/4)	(72/16/23/5)	(25/11/19/4)	(16/10/12/4)
Ethnicity (% C/A/AC/O)	(80.6/10.2/4.5/4.5)	(62/12.1/19.8/4.3)	(42.4/19/32.8/6.8)	(38.1/23.8/28.6/9.5)
Hydroxychloroquine (%)	ND	22 (18.9)	8 (13.5)	3 (7.14)
Pred<10mg/day ± hcq (%)	ND	16 (15.1)	26 (44.06)	13 (30.9)
Pred>10mg/day ± hcq (%)	ND	5 (4.3)	6 (10.17)	8 (19.04)
Pred + IS ± hcq (%)	ND	65 (56.03)	19 (32.2)	18 (42.86)

Patients fulfilling the revised classification criteria for SLE were assessed for disease activity with the British Isles Lupus Assessment Group Index (BILAG)[51,52]. The BILAG index is a clinical measure of disease that distinguishes activity in nine different organ systems[53]. Each organ system was given a grade A, B, C, D or E, where A was the most active and E was least active. In this study, the grades were converted into numerical scores using the BILAG-2004 index, whereby A=12, B=8, C=1, D=0 and E=0[54]. Following this, global BILAG scores were calculated by adding the sum of the values from all organ systems. Patients with a global score higher than 8 were considered active. All patients treated with rituximab therapy had active disease prior to treatment, after which they were either responding (BILAG<8; responders) or not responding (BILAG >8; non-responders) upon repopulation of B cells. The patients in this study were grouped as SLE patients not treated with rituximab, responders or non-responders to rituximab therapy. The following abbreviations were used: Caucasian (C), Asian (A), African Carribean (AC), Other (O), prednisolone (Pred), Hydroxychloroquine (hcq), IS (immunosuppressants).

Supplementary Experimental Procedures

Cell Culture. PBMCs and isolated cell populations were cultured in RPMI 1640 containing L-glutamine and $NaHCO_3$ (Sigma-Aldrich) supplemented with 10% FCS (Biosera) and 100 IU/ml penicillin and streptomycin (Sigma-Aldrich). CD154 transfected CHO cells and untransfected control cells (kindly provided by Prof. R. Mageed, Queen Mary University, London) were grown in Dulbecco's Modified Eagle's Medium (DMEM; Sigma-Aldrich) containing 4500 mg/ml glucose, 110 mg/ml sodium pyruvate and 2mM L-glutamine, supplemented with 5% FCS and 100U/ml penicillin and streptomycin in sterile culture flasks. The CHO cells received gamma-irradiation via a caesium source for 10 minutes (250 kV, 11.6 mA, 7Gy/min) with minimal covering of culture media prior to use in co-culture experiments, with a B cell:CHO cell ratio of 10:1.

Flow Cytometry. B cells were stained with CD19-PECy7 (Biolegend), CD24-APCCy7, CD38-PerCPCy5.5, CD27-PETexRed, IgD-eFluor450, IgM-FITC and CD138-PE (eBioscience). pDCs were stained with CD123-eFluor450, CD80-PerCPCy5.5, CD86-PerCPCy5.5, CD83-PE, CD40L-APC (eBioscience), BDCA-2-FITC (Miltenyi Biotech). For analysis of intracellular cytokine production, cells were stimulated with 0.5μg/ml plate-bound anti-CD3 mAb (Hit3a) (BD Biosciences) or 1μM CpG ODN2395 (CpGC) for 3 days, or with 1μM CpG ODN2116 (CpGA) (InvivoGen) for 20 hr. Cells were washed, fixed, permeabilized and stained for detection of intracellular cytokines IL-10-APC (BD Biosciences), TNFα-eFluor450, IL-6-PE and IFNγ-PE

(eBiosciences), and IFNα-APC (Miltenyi Biotech); or for Ki67-PE, Blimp1-APC and Pax5-BV510 (BD Biosciences).

B cell differentiation assay. Isolated $CD19^+$ B cells, or FACS sorted B cell subsets were cultured either alone or with BDCA-$2^+$$CD123^+$ pDCs (unless otherwise stated at 1:3 pDC:B cell) and stimulated with 1μM of CpGC, CpGB, CpGA, or HCMV lysates (1/100 dilution; kindly provided by Prof. A. Akbar, UCL) for 3 or 10 days . 50ng/ml PMA (Sigma), 250ng/ml ionomycin (Sigma) and GolgiPlug (BD Biosciences) were added for the last 5 hr of culture, after collecting supernatants. After 3 days, cells were fixed and permeabilized with intracellular staining reagents according to manufacturer's instructions (eBioscience) and stained with anti-IL-10, TNFα and IL-6. IL-10 in the supernatant was measured by ELISA (R&D Systems). After 10 days, IgG and IgM were measured in the supernatants from the cells cultured by ELISA (Sigma). Neutralizing antibodies against IFNα (PBL), IFNα/βR2 (PBL) and CD40L (BD Biosciences) were used in the co-culture experiments; or recombinant IFNα (PBL) or IFNβ were added to isolated $CD19^+$ B cells. For experiments with SLE PBMCs, isolated B cells were co-cultured with pooled allogeneic pDCs from either healthy individuals or SLE patients for 3 days with CpGA, followed by intracellular detection of IL-10. Supernatants from pDCs cultured with CpGA were measured for cytokines IL-1β, IL-6, IL-10, IL-13, IL-17a and TNFα by cytometric bead array (BD Biosciences) and IFNα by ELISA (PBL). PBMCs from healthy individuals or SLE patients were either undepleted or depleted of pDCs, and stimulated with CpGC for 72 hr.

B cell functional assays. CD19$^+$ B cells or flow cytometry sorted B cell subsets were cultured with CpGC, either alone or with pDCs for 48 hr. CD19$^+$ B cells or CD24$^+$CD38^{hi} and CD24$^-$CD38^{hi} B cell subsets were then re-isolated by FACS and co-cultured 1:1 with freshly isolated CD4$^+$CD25$^-$ T cells for 72 hr. PMA, ionomycin and GolgiPlug were added during the last 5 hr of culture. Cells were surface stained, permeabilized and stained for intracellular expression of TNFα, IFNγ and IL-10. Alternatively, B cell subsets isolated by flow cytometry were co-cultured with irradiated CD40L-expressing CHO cells or control CHO cells (1:10 CHO:B cells) for 72 hr. Supernatants from the cell cultures were then transferred to freshly purified autologous pDCs, and stimulated with CpGA for 20 hr. GolgiPlug was added for the last 5 hr, and cells were permeabilized and stained for the detection of intracellular IFNα. Neutralizing antibodies against IL-10 (R&D System), and IL-10R (BD Biosciences) were used in the co-culture experiments.

Statistical analysis.

Data were analyzed as followed:

Figure 1: two-way ANOVA (A and E), paired *t*-test (C), one-way ANOVA (D and F).

Figure 2. two-way ANOVA.

Figure 3. one-way ANOVA (A, B, D-F), two-way ANOVA (C).

Figure 4. one-way ANOVA (B-E), Pearson's correlation (F-H).

Figure 5. paired *t* test (A), one-way ANOVA (B and C), unpaired t-test (D-H), two-way ANOVA (I and J).

Figure 6. unpaired *t* test (A and G), one-way ANOVA (B-E).

Figure 7. one-way ANOVA (H and I).
